# Vorinostat Potentiates Chemoimmunotherapy in Immune‐Enriched Pancreatic Cancer

**DOI:** 10.1002/advs.202521844

**Published:** 2026-04-13

**Authors:** Chen Chen, Dingru Li, Yingna Liao, Zehua Wang, Chunbin Zhu, Zifeng Zhang, Liquan Jin, Yueyue Chen, Jiaoshun Chen, Junyi Xu, Miaoyan Wei, Rong Tang, Xianjun Yu, Si Shi

**Affiliations:** ^1^ Department of Pancreatic Surgery Fudan University Shanghai Cancer Center Shanghai China; ^2^ Department of Oncology Shanghai Medical College Fudan University Shanghai China; ^3^ Shanghai Pancreatic Cancer Institute Shanghai China; ^4^ Shanghai Key Laboratory of Precision Medicine for Pancreatic Cancer Shanghai China; ^5^ Department of Oncology The First Affiliated Hospital of Zhengzhou University Zhengzhou China

**Keywords:** chemoimmunotherapy, immune‐enriched, metabolic reprograming, PDAC, SAHA

## Abstract

Although pancreatic ductal adenocarcinoma (PDAC) is generally considered an immunologically “cold” tumor, approximately 20% of cases can be classified as immune‐hot. However, this immune‐enriched (IE) phenotype does not confer a significant survival advantage, highlighting the need to investigate its underlying mechanisms and identify effective therapies. By integrating in vitro drug screening and in silico sensitivity prediction, we identified the HDAC inhibitor vorinostat (SAHA) as a potent sensitizer to chemoimmunotherapy specifically in the IE‐PDAC. This effect was validated using T cell‐organoid co‐cultures and patient‐derived xenografts with humanized immune systems. Mechanistically, abundant cytokines (TNF‐α, FGF) in the IE tumor microenvironment promote FASN and PARP9 expression. This leads to free fatty acid accumulation and enhanced oxidative phosphorylation, supporting tumor cell survival. SAHA disrupts this “metabolic trap” by concurrently suppressing FASN and PARP9. Single‐cell RNA sequencing revealed that the Gemcitabine‐SAHA combination remodels the tumor microenvironment by enhancing CD8^+^ T cell function and depleting cancer‐associated fibroblasts. Clinically, we defined a CD8^high^/FASN^high^/PARP9^high^ signature that identifies an IE patient subgroup with poor survival, representing those most likely to benefit from the “Gemcitabine‐Nivolumab‐SAHA” triple‐combination therapy.

## Graphical abstract

Immune‐enriched pancreatic cancer does not confer a significant survival advantage. SAHA sensitizes these “hot” tumors to chemoimmunotherapy by disrupting a FASN/PARP9‐driven “metabolic trap” and enhancing CD8^+^ T cell function. A CD8^high^/FASN^high^/PARP9^high^ signature identifies patients who are most likely to benefit from the “gemcitabine‐nivolumab‐SAHA” triple combination.

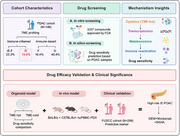

## 1 Introduction

Pancreatic ductal adenocarcinoma (PDAC) remains a highly lethal malignancy, with a global five‐year survival rate below 10% [1]. Gemcitabine continues to serve as the backbone of first‐line chemotherapy in PDAC, yet its clinical benefit remains constrained by intrinsic and acquired resistance mechanisms [2, 3, 4]. A key driver of PDAC aggressiveness is its complex tumor microenvironment (TME), which is often classified as “immune‐desert” characterized by sparse T‐cell infiltration and a dominant stroma that restricts immunotherapy efficacy [5, 6].

Contrary to this prevailing view, a substantial subset of PDAC patients (approximately 20%) present with an “immune‐enriched” (IE) or “TME‐hot” phenotype, as identified by recent studies [7, 8]. However, unlike in most other cancers where immune infiltration predicts improved outcomes, these PDAC patients do not show a significant survival advantage [9, 10]. This paradox highlights the presence of uncharacterized intrinsic resistance mechanisms that undermine therapeutic efficacy even in a seemingly favorable immune context. Hence, we propose a core scientific theory: a distinct “metabolic trap” driven by the IE‐TME may be responsible for IE‐PDAC's unfavorable prognosis and the epigenetic modulator vorinostat (SAHA) has the potential to break this trap.

The complex interplay between immune activation and metabolic reprogramming represents a crucial axis of prognosis in PDAC. A recent study has stratified PDAC into lipomet‐ and glucomet‐subtypes, each exhibiting differential drug sensitivity correlated with their metabolic phenotypes [11]. Moreover, neoadjuvant chemotherapy has been shown to remodel the PDAC TME, not only driving a transition from an “immune‐cold” to an “immune‐hot” state characterized by increased CD8^+^ T‐cell infiltration but also shifting cellular metabolism from glycolysis toward lipid metabolism preference [12]. This coordinated remodeling implies that the IE‐TME coincides with a specific lipid‐metabolic state. However, such a lipid‐metabolic preference might in turn dampen antitumor immunity. Supporting this notion, a study in hepatocellular carcinoma (HCC) revealed that Riplet deficiency promotes a lipid‐metabolic activation that directly induces T‐cell exhaustion and confers resistance to anti‐PD‐1 therapy [13]. Therefore, we posit the IE‐TME might boost lipid metabolism, which crippled cytotoxic T‐cell function and weakened IE‐PDAC's treatment response.

Metabolic and immune remodeling within the TME is both regulated by epigenetic modulation. Epigenetic regulation, particularly through histone acetylation, might serve as a central mechanism orchestrating the immune‐metabolic loop in PDAC. As a well‐established cancer hallmark, epigenetic dysregulation is fundamentally governed by the dynamic balance of histone acetyltransferases (HATs) and histone deacetylases (HDACs) [14, 15]. HDACs act at the nexus of tumor‐immune crosstalk [16]. Specifically, HDACs promote immune evasion by modulating the expression of immune checkpoint molecules (e.g., PD‐L1) and antigen‐presenting machinery (e.g., MHC) on tumor cells [17, 18], as well as by directly driving the exhaustion of effector T cells [19]. Moreover, evidence positions HDACs as regulators of metabolism reprogramming, exemplified by findings that nuclear VCP promotes fatty acid oxidation (*FAO*) gene transcription via HDAC1 degradation to fuel cancer cells in glucose‐restricted conditions [20]. Accordingly, our study found that SAHA can limit fatty acid metabolism in IE‐PDAC and thus enhance the efficacy of chemoimmunotherapy.

Here, by integrating an in vitro drug screening platform with an in silico prediction pipeline, we identified SAHA as a potent chemoimmunotherapy sensitizer in IE‐PDAC, which acts by concurrently orchestrating lipid metabolism and remodeling the immune microenvironment. This chemo‐targeted‐immunotherapy strategy holds promise for improving survival outcomes in this challenging PDAC subgroup.

## 2 Material and Methods

### 2.1 Clinical Pancreatic Cancer Specimens

Tissue microarrays (TMA) were established including 299 pancreatic cancer tissues from the Fudan University Shanghai Cancer Center (FUSCC) cohort. All samples were obtained from patients who underwent surgical resection without prior treatment and were pathologically verified as PDAC between October 1, 2020, and August 31, 2021. The study was conducted with the approval of the FUSCC Ethics Committee (2506‐Exp213), and written informed consent was obtained from each patient.

### 2.2 Cell Lines and Cell Culture

PDAC cell lines Panc‐1 (RRID: CVCL_0480) and Mia‐PaCa2 (RRID: CVCL_0428) were obtained from the American Type Culture Collection (ATCC). KPC‐0116 cells were derived from *Kras* ^LSL‐G12D/+^; *Trp53* ^LSL‐R172H/+^; *Pdx1‐Cre* (KPC) mice and cultivated in our laboratory in vitro as we previously described [12]. The human pancreatic cancer‐associated fibroblast (CAF) cell line was isolated from PDAC tissue and expanded in vitro in our laboratory. All cell lines were regularly confirmed to be free of mycoplasma contamination using the GMyc‐PCR Mycoplasma Test Kit (Yeasen Biotech, Cat# 40601ES20). Panc‐1, Mia‐PaCa2, and KPC‐0116 cells were cultured in DMEM and CAFs were cultured in DMEM/F12. All media were supplemented with 10% fetal bovine serum (LONSERA, Shuangru Biotechnology, Cat# S711‐050S) and 1% penicillin‐streptomycin at 37°C in a 5% CO_2_ incubator. Cell line authentication was performed by short tandem repeat (STR) profiling.

### 2.3 PBMC Extraction and Activation

Peripheral blood mononuclear cells (PBMCs) were isolated from blood samples of 160 PDAC patients using a human lymphocyte separation tube (Dakewe Biotech, Cat# 7922112). Isolated PBMCs were activated with anti‐human CD3/CD28 (Stemcell, Cat# 10971) and 30 U/mL recombinant human IL‐2 (MCE, Cat# HY‐P7037) for 24 h prior to co‐culture.

### 2.4 Elisa

Levels of TNF‐α and IFN‐γ in supernatants from cocultures of PBMCs with Panc‐1 cells were measured by ELISA (Cloud‐Clone, Cat# USEA133Hu, Cat# USEA049Hu) to determine the cell ratio that best recapitulates an “immune‐hot” cytokine milieu. Cocultures were established at varying PBMC‐to‐tumor cell ratios (0:1, 0.5:1, 1:1, 2:1, 5:1, 10:1, and 20:1) (Figure S1A). The assay was performed according to the manufacturer's protocol.

### 2.5 Immune‐Hot Mimicry

Activated PBMCs were directly co‐cultured with PDAC cell lines at a 5:1 ratio for 48 h. Following co‐culture, the supernatant was collected and centrifuged at 3000 rpm for 10 min to remove cellular debris. The resulting cell‐free supernatant was designated as the “immune‐hot” mimicry conditioned medium and used as the base medium for subsequent in vitro drug screening and functional assays. Cytokine screening (Listed in Table S1) was performed with a concentration of 10 ng/mL.

### 2.6 Patient‐Derived PDAC Organoids (PDO)

Patient‐derived organoids (PDOs) were established from 21 fresh PDAC tissues. Tissue fragments were digested in Tumor Tissue Digestion Solution (bioGenous, Cat# K601003) at 37°C for 1 h with gentle rotation. The resulting cell suspension was filtered through a 70 µm strainer, centrifuged at 300 × g for 5 min, and treated with Red Blood Cell Lysis Solution (Beyotime Biotechnology, Cat# C3702) for 5 min. The pellet was resuspended in Matrigel (Corning, Cat# 356231), and 40 µL aliquots were seeded per well in a 48‐well plate. After polymerization at 37°C for 20 min, each well was overlaid with 400 µL of Pancreatic Cancer Organoid Basal Medium (bioGenous, Cat# K2101‐PC) for continued culture. PDOs and activated PBMCs were co‐cultured at a 1:5 ratio in OrganoidpleX medium (bioGenous, Cat# CO1233) on Matrigel‐coated 96‐well plates. Apoptosis was subsequently assessed by staining with a green‐fluorescent caspase 3/7 probe (Invitrogen, Cat# R37111).

### 2.7 Drug Response In Vitro Testing

A library of 3,337 FDA‐approved small molecules (Selleck, Cat# L3800) was utilized for high‐throughput drug screening. For conventional 2D cell cultures, cell viability was assessed using the Cell Counting Kit‐8 (CCK‐8, Sparkjade, Cat# CT0001) according to the manufacturer's protocols. Cells were seeded in 96‐well plates, treated with different compounds, and absorbance was measured at 450 nm using a microplate reader.

PDO viability following drug treatments was assessed using the LivingCell‐Fluo Organoid Vitality Assay Kit (bioGenous, Cat# E238004). This fluorescence‐based assay quantifies viability by measuring cellular metabolic activity. In this assay, viable cells reduce the non‐fluorescent probe in the kit to a red‐fluorescent product (Excitation/Emission: 560/590 nm), whereas non‐viable cells lose this metabolic capacity and generate no fluorescent signal.

### 2.8 Animal Experiments

BALB/c nude, C57BL/6J, and NOD‐Prkdc‐scid Il2rg‐/‐ (NPSG) mice (female, 6 weeks old, 18–20 g) were maintained under specific pathogen‐free (SPF) conditions at the Shanghai Cancer Center. All animal procedures were approved by the Institutional Animal Care and Use Committee (IACUC) and conducted in accordance with NIH guidelines (FUSCC‐IACUC‐2024490, FUSCC‐IACUC‐2025147).

#### 2.8.1 Subcutaneous and Orthotopic Models

For the subcutaneous tumor model, 0.5 × 10^6^ KPC cells were inoculated into the left flank of each mouse. Tumor volume was measured every three days using a caliper and calculated as (length × width^2^) / 2. For the orthotopic model, the spleen was exteriorized through a left flank incision. The pancreatic tail, which lies adjacent to the spleen, was carefully everted using a sterile cotton swab to achieve clear exposure. Subsequently, 0.5 × 10^6^ KPC cells were injected along the longitudinal axis of the pancreas under direct vision. Tumor growth was monitored weekly via bioluminescence imaging, and the relative tumor volume was quantified. In both models, SAHA (75 mg/kg, Selleck, Cat# S1047) was administered daily by oral gavage on a 5‐days‐on/2‐days‐off weekly schedule. Gemcitabine (37.5 mg/kg, Selleck, Cat# S1714) was delivered intraperitoneally once every 4 days.

#### 2.8.2 Humanized Patient‐Derived Xenograft (PDX) Model

To better model the human TME for therapeutic evaluation, we employed a humanized PDX model. Fresh tumor tissues from treatment‐naïve PDAC patients were implanted subcutaneously into NPSG immunodeficient mice to establish F0 lines. PDXs were expanded through serial passage (F1 to F3). For humanization, F3 tumor‐bearing mice received 7 × 10^6^ human PBMCs (Donor ID: P123050709C) intravenously one week after tumor implantation, with successful immune reconstitution monitored weekly via flow cytometry quantification of human CD45^+^ (hCD45^+^) cells in peripheral blood. This humanized PDX model preserves the original tumor's histopathological architecture and stromal components while enabling the study of drug responses within a humanized immune context.

Once stable humanization was established, PDXs were randomized into the following treatment groups: vehicle control, gemcitabine combined with nivolumab (GN), SAHA, and the triple combination of gemcitabine, nivolumab, and SAHA (GNS). SAHA was administered daily by oral gavage at 5 mg/kg on a schedule of 5 consecutive days followed by a 2‐day break each week. Gemcitabine was delivered intraperitoneally at a dose of 1 mg/kg once every 4 days. Nivolumab (1 mg/kg, Selleck, Cat# A2002) was administered intraperitoneally once per week. Tumor volumes and body weights were monitored regularly every three days.

### 2.9 Construction of Stable Cell Lines

Stable PARP9‐knockdown cell lines were generated in both Panc‐1 and Mia‐PaCa2 cells using lentiviral transduction. Cells were transduced with either control (empty vector) or PARP9‐specific shRNA lentiviral particles. After 24 h of transduction, the viral supernatant was replaced with fresh complete medium. Selection was initiated 72 h post‐transduction using puromycin at a concentration of 1 µg/mL. Stable polyclonal populations were maintained under continuous puromycin selection for 72 h.

### 2.10 RT‐qPCR Analysis

Total RNA was extracted using TRIzol reagent (Invitrogen, Cat# 15596026cn) and reverse‐transcribed into cDNA using the PrimeScript RT Reagent Kit (Vazyme, Cat# R333). Quantitative PCR was performed on a QuantStudio system using SYBR Green master mix (Vazyme, Cat# Q711). Gene expression was normalized to GAPDH and analyzed using the 2^(‐ΔΔCt) method.

### 2.11 Western Blot and Coimmunoprecipitation (Co‐IP)

Total protein was extracted from cells using RIPA lysis buffer (NCM Biotech, Cat# WB3100) with a 1% protease and phosphatase inhibitor cocktail (NCM Biotech, Cat# P002). Nuclear and cytoplasmic proteins were extracted using NE‐PER Nuclear and Cytoplasmic Extraction Reagent (Thermo, Cat# 78833).

Proteins were quantified with a BCA Protein Assay Kit (Epizyme Biotech, Cat# ZJ102). Equal amounts of protein lysates were resolved by SDS‐PAGE and transferred onto PVDF membranes (Millipore, Cat# ISEQ00010). After blocking with 5% bovine serum albumin (BSA), the membranes were incubated overnight at 4°C with primary antibodies, followed by incubation with HRP‐conjugated secondary antibodies for 1 h at room temperature. Immunoreactive bands were visualized using an enhanced chemiluminescence kit (Epizyme Biotech, Cat# SQ201) and captured on X‐ray film.

For Co‐IP assays, cells were lysed in NP‐40 buffer supplemented with a protease inhibitor cocktail (NCM Biotech, Cat# P002). Cell lysates were incubated with the indicated antibodies for 2 h at 4°C, followed by incubation with Protein A/G magnetic beads (ACE Biotechlogy, Cat# MP1001) overnight at 4°C. The immunoprecipitated complexes were washed, eluted, and subsequently analyzed by Western blot or subjected to mass spectrometry for protein identification. The details of the antibodies used are listed in Table S2.

### 2.12 Flow Cytometry Analysis

Single‐cell suspensions from mouse tissues and PBMCs were obtained. To minimize nonspecific antibody binding, cells were initially blocked with an anti‐mouse CD16/32 antibody (BioLegend, Cat# 101320) for 10 min on ice. For surface marker staining, cells were incubated with fluorochrome‐conjugated antibodies (listed in Table S3) for 45 min in the dark at 4°C. For intracellular staining, cells were fixed and permeabilized using the Cytofix/CytopermTM Fixation/Permeabilization kit (BD Biosciences, Cat# 554714), followed by incubation with antibodies against intracellular targets for 45 min at 4°C. After final washing, cells were resuspended in staining buffer and analyzed immediately on a Beckman CytoFlex S flow cytometer. Data was analyzed using FlowJo software.

### 2.13 CUT & Tag Assay

The CUT&Tag assay was performed with the technical assistance of Jiayin Biomedical Technology (Shanghai, China) using the NovoNGS CUT&Tag High‐sensitivity Kit (Novoprotein, Cat# N259‐YH01). Briefly, Panc‐1 cells cultured under “immune‐hot” mimetic conditions were harvested and incubated with primary antibodies against HDAC1, HDAC2, and HDAC3 (Table S2). Sequencing libraries were constructed and sequenced on an Illumina platform. For bioinformatic analysis, raw sequencing reads were processed with Trimmomatic to remove adapter sequences and low‐quality bases [21]. Clean reads were aligned to the reference genome, and fragment size distributions were calculated from the properly paired reads in the resulting BAM files. Peak calling was performed using MACS2, and significant peaks were annotated with bedtools [22].

### 2.14 Mitochondrial Respiration Analysis

Mitochondrial respiratory function was assessed by measuring the oxygen consumption rate (OCR) using the Seahorse XFe Extracellular Flux Analyzer (Agilent) with the XF Cell Mito Stress Test Kit (Agilent, Cat# 103015–100). Cells were seeded into XF96 microplates and incubated overnight at 37°C. On the day of assay, the culture medium was replaced with 50 µL of assay medium (Seahorse XF DMEM Base Medium containing 1 mM sodium pyruvate, 1 mm glutamine, and 1 mm glucose) per well, followed by addition of another 130 µL of assay medium. The plate was then incubated for 60 min at 37°C in a non‐CO_2_ incubator for temperature and pH equilibration. The mitochondrial stress test was performed with sequential injections of metabolic modulators: 1.5 µm oligomycin (ATP synthase inhibitor), 1.0 µm FCCP (mitochondrial uncoupler), and 0.5 µm rotenone/antimycin A (complex I and III inhibitors). OCR measurements were taken at baseline and following each injection to evaluate key parameters of mitochondrial function.

### 2.15 Immunofluorescence (IF) Staining

For immunofluorescence analysis, cells or tissue specimens were fixed in 4% paraformaldehyde, permeabilized with 0.2% Triton X‐100 (Sigma, Cat# T8787) and incubated overnight at 4°C with the indicated primary antibodies (Table S4). After washing, samples were incubated with species‐matched secondary antibodies for 1 h at room temperature. Nuclei were visualized using DAPI‐containing ProLong Diamond Antifade Mountant (Thermo, Cat# P36966). Fluorescence images were acquired using a Zeiss fluorescence microscope.

### 2.16 Multiplex Fluorescent Immunohistochemistry (mIHC)

Formalin‐fixed, paraffin‐embedded pancreatic cancer specimens from FUSCC were sectioned at 3 µm thickness and mounted on glass slides. After baking at 65°C for 2 h, slides were deparaffinized in xylene and rehydrated through a graded ethanol series. Antigen retrieval was conducted in sodium citrate buffer (pH 6.0) using microwave heating, followed by inhibition of endogenous peroxidase activity with 0.3% H_2_O_2_ in methanol. Sections were blocked with goat serum for 30 minu and then incubated with primary antibodies (Table S4) for 1 h at room temperature. After TBST washing, horseradish peroxidase‐conjugated secondary antibodies were applied, and signals were developed using the kit's fluorescent dye reagents. This staining cycle, including antigen retrieval, primary antibody incubation, and signal development, was repeated sequentially for each marker. Finally, nuclei were counterstained with DAPI, and slides were mounted with anti‐fade reagent. Image acquisition was performed using a Zeiss fluorescence microscope.

### 2.17 Immunohistochemistry (IHC) Staining

Tissue samples were fixed in 4% formaldehyde overnight at room temperature and embedded in paraffin. Sections (4 µm) were deparaffinized in xylene, and endogenous peroxidase activity was quenched with 3% hydrogen peroxide. Antigen retrieval was performed using EDTA (Sigma, Cat# E9884) under heat induction. The sections were then incubated with primary antibodies (Table S4) at 4°C overnight. Immunostaining was performed using the Dako REAL EnVision detection system (DAKO, Cat# K5007) according to the manufacturer's protocol. Stained images were acquired with an Olympus BX61 microscope.

### 2.18 Cell Proliferation (CCK‐8) Assay

CAFs were plated in 96‐well plates at 1000 cells per well in 100 µL of complete culture medium and allowed to adhere for 24 h. Cells were then treated with GEM (2 nm), SAHA (10 nm), the combination of both, or left untreated as a control. Cell viability was assessed at the indicated time points (24, 48, 72, 96, 120, and 144 h) after seeding using the Cell Counting Kit‐8 (Sparkjade, Cat# CT0001). At each time point, 10 µL of CCK‐8 reagent was added to each well, followed by incubation at 37°C for 2 h. Absorbance was then measured at a wavelength of 450 nm.

### 2.19 Colony Formation Assay

CAFs were seeded in 6‐well plates at a density of 5000 cells per well and allowed to adhere for 24 h. Following treatment with GEM (1 nm), SAHA (5 nm), the combination of both, or untreated control, cells were cultured for 9 days. Subsequently, cells were fixed with 4% paraformaldehyde and stained with 0.1% crystal violet. Colony images were acquired using a camera.

### 2.20 Statistical Analysis

All statistical analyses were performed using GraphPad Prism 9.5 and R software (4.3.3). Data from at least three independent biological replicates are presented as mean ± standard deviation (SD). For continuous variables, when the assumptions of normality (assessed by the Shapiro–Wilk test) and homogeneity of variances (assessed by Levene's test) were met, an unpaired Student's *t*‐test was used; otherwise, the non‐parametric Mann–Whitney U test was applied. For comparisons among multiple independent groups, a one‐way ANOVA was conducted, followed by Tukey's post‐hoc test. For experiments with a two‐factor factorial design, a two‐way ANOVA was performed to assess main and interaction effects. Survival analysis was conducted using the log‐rank test. For analyzing IE‐TME signatures (CD8/FASN/PARP9), samples were stratified into low‐ and high‐expression groups using the median value as the cut‐off. All *p*‐values were calculated using two‐tailed tests. A *p*‐value <0.05 was considered statistically significant, with significance levels denoted as ^*^ *p* < 0.05, ^**^ *p* < 0.01, ^***^ *p* < 0.001, and ^****^ *p* < 0.0001.

## 3 Results

### 3.1 Integrated Drug Screening Identifies SAHA as a Potentiator of Gemcitabine Efficacy in Immune‐Enriched PDAC

**FIGURE 1:**
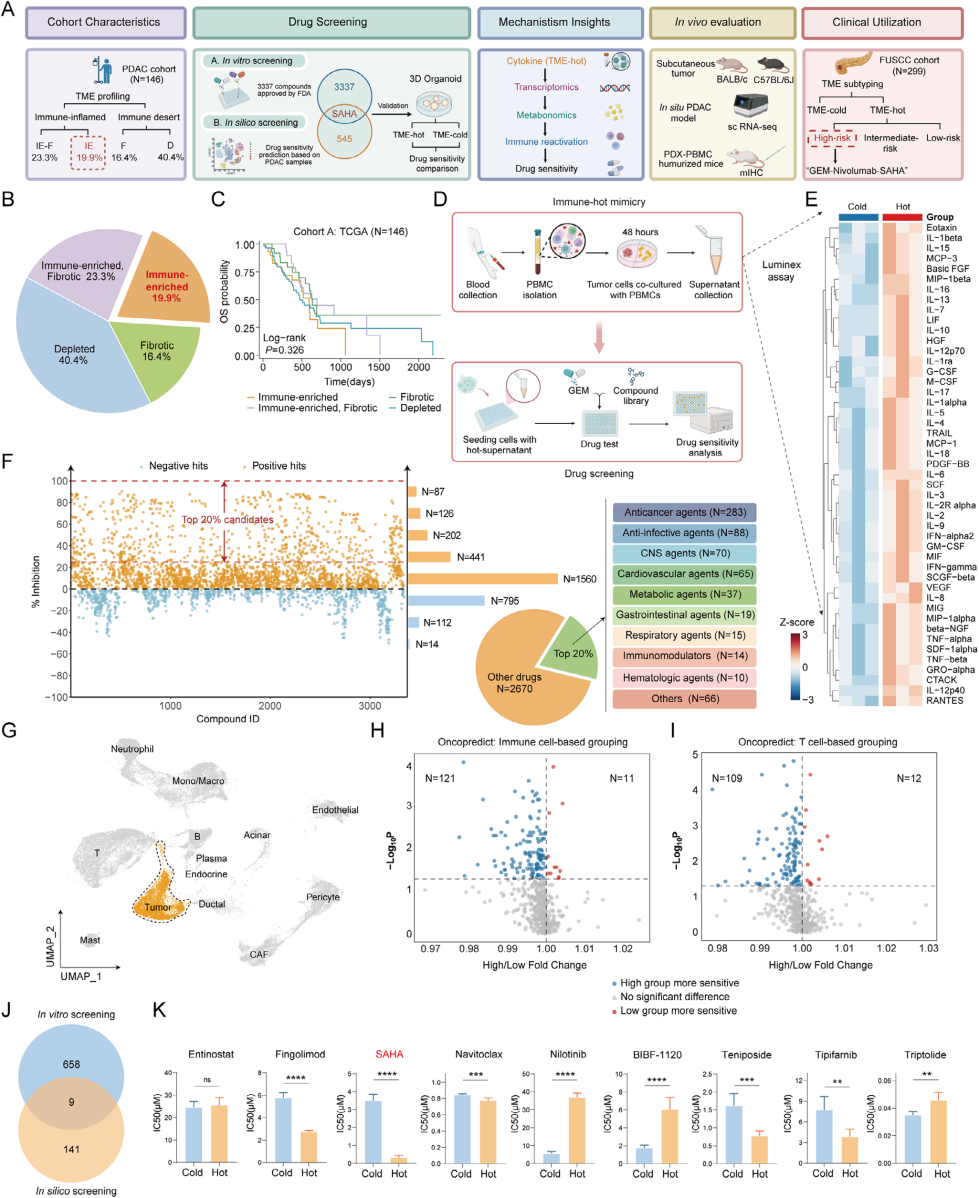
Integrated Drug Screening Identifies SAHA for Combination Therapy in Immune‐Enriched PDAC. (A) Schematic of the multi‐platform study design integrating drug screening, functional validation, mechanistic insights, and clinical correlation analysis. (B) Classification of the TCGA‐PAAD cohort (*n* = 146) into four distinct TME subtypes: Immune‐Enriched (IE), Immune‐Enriched Fibrotic (IE‐F), Fibrotic (F), and Depleted (D). (C) Kaplan–Meier survival curves of PDAC patients stratified by TME subtype. (D) Schematics of the in vitro screening workflow. (E) Heatmap of Luminex data confirming the success of in vitro “immune‐hot” mimicry. (F) Visualization of the primary screen results. The scatter plot highlights the top 20% potentiation hits, and the pie chart shows their functional classification. (G) UMAP plot of scRNA‐seq data in PDACs (N = 41), with the tumor cell cluster highlighted in orange. (H,I) OncoPredict analysis of differential drug sensitivity (IC50 fold‐change) between defined subgroups: immune‐high vs. immune‐low (H) and T cell‐high vs. T cell‐low (I). (J) Venn diagram showing overlap between top hits from in vitro (*n* = 667) and in silico (*n* = 150) screens. (K) Validation of the 9 shortlisted candidates. Bar graph compares the IC50 values (*n* = 6) of each compound in combination with gemcitabine (500 nm) between “immune‐cold” (blue) and “immune‐hot” (orange) mimetic conditions. Data were represented as the mean ± SD. Statistical significance was determined by the log‐rank test (C) and by unpaired two‐tailed Student's *t*‐tests (K). ^**^, *p* < 0.01; ^***^, *p* < 0.001; ^****^, *p* < 0.0001; ns, not significant.

To identify compounds that potentiate efficacy in IE‐PDAC, we conducted a high‐throughput screening of 3337 FDA‐approved agents using an integrated in vitro and in silico approach. A schematic overview of the drug screening pipeline is presented in Figure 1A. We evaluated the clinical relevance of TME subtypes by applying a previously established TME classification to the TCGA‐PAAD cohort [7]. Patients were categorized into four TME subtypes: Immune‐Enriched (IE), Immune‐Enriched Fibrotic (IE‐F), Fibrotic (F), and Depleted (D) (Figure 1B). Surprisingly, Kaplan–Meier analysis revealed that patients with the IE subtype did not exhibit a significant survival advantage over those in other subtypes (log‐rank *p* >0.05; Figure 1C).

To simulate the IE‐TME in vitro for drug screening, we generated a conditioned medium by co‐culturing PBMCs with Panc‐1 cells (5:1 ratio) for 48 h (Figure 1D), with the ratio optimized by cytokine assessment via ELISA (Figure S1A). This medium, containing abundant immune‐regulatory cytokines as confirmed by Luminex assay and proteomic analyses (Figure 1E; Figure S1B–D), was defined as the “immune‐hot‐mimicry” medium. Subsequently, this “immune‐hot‐mimicry” medium was used to screen the compound library for agents that enhanced gemcitabine efficacy. To mitigate batch effects across independent experiments, gemcitabine monotherapy was used as an internal reference in each screen. Cell viability was assessed via CCK‐8 assay, with the effect of each drug combination normalized to that of gemcitabine alone. Among the 3,337 compounds screened, 2,416 potentiated gemcitabine's cytotoxicity, from which the top 20% (667 compounds) were selected for further investigation (Figure 1F).

In parallel, we performed an in silico drug screen using single‐cell RNA sequencing (scRNA‐seq) data from 41 PDAC patients (Figure 1G; Figure S1E). To preclude confounding effects from non‐malignant cells on drug sensitivity prediction, we subset tumor cell transcriptomics for drug sensitivity prediction. First, samples were stratified into two groups based on the proportion of overall immune cells or T cells (Figure S1F,G). Second, by applying the OncoPredict algorithm to tumor cell‐specific transcriptomes [23], we inferred differential drug sensitivity between the groups and identified 150 compounds with subtype‐specific efficacy and superior drug response in “immune‐hot” over “immune‐cold” microenvironments (Figure 1H,I).

Integration of the top hits from both screens, 667 from in vitro screening and 150 from in silico analysis, yielded a shortlist of 9 overlapping compounds showing distinct relative IC50 values across “cold” and “hot” tumor subtypes (Figure 1J; Figure S1H,I). Subsequent dose‐response validation performed in “hot‐mimetic” conditioned medium determined the combination IC50 values for each candidate when co‐administered with a fixed dose (500 nm) of gemcitabine (Figure 1K).

### 3.2 Functional Validation in 3D PDOs Confirms Enhanced Efficacy of Gemcitabine‐SAHA (G‐S) Combination in Immune‐Enriched Contexts

**FIGURE 2:**
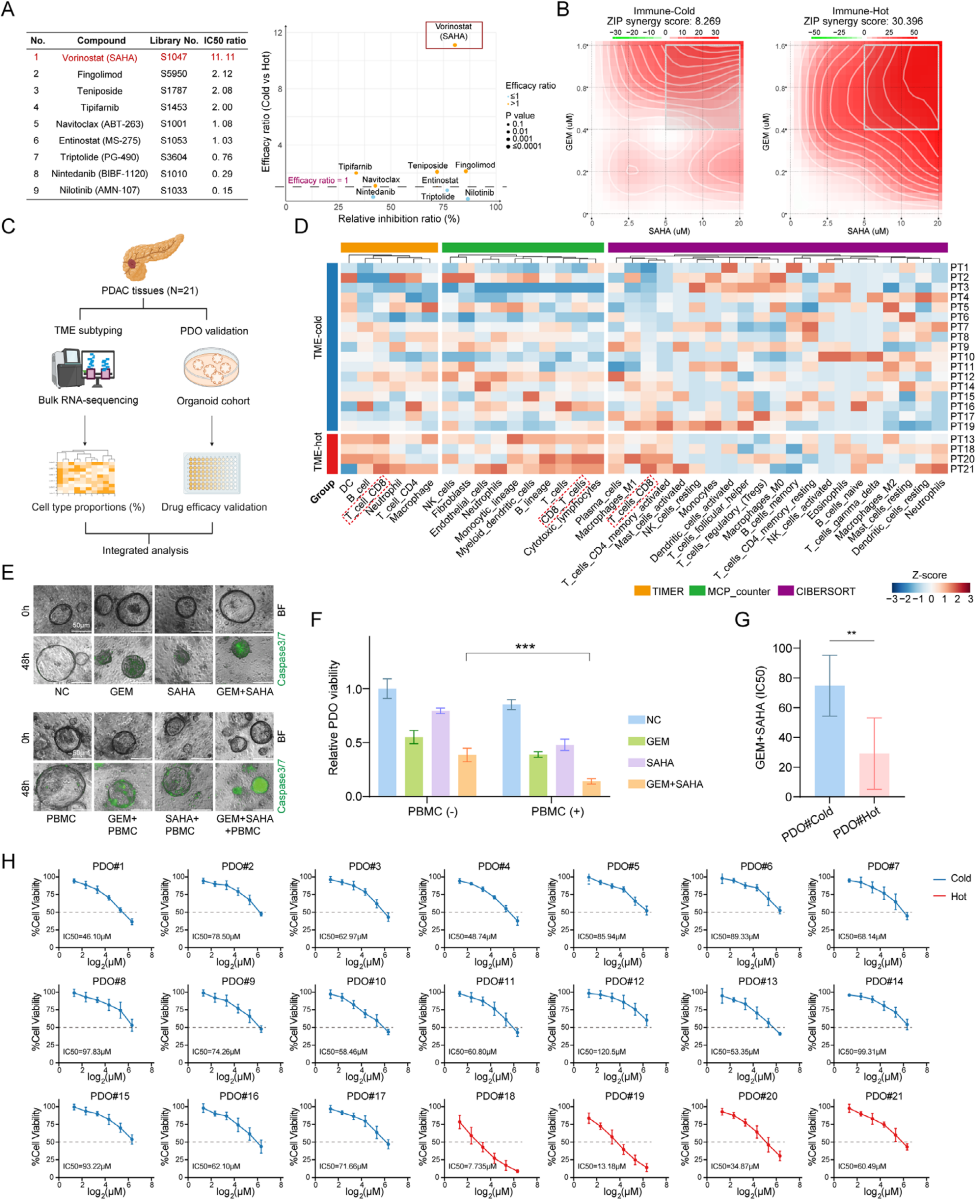
SAHA Demonstrates Superior Synergistic Activity with Gemcitabine in Immune‐Enriched PDOs. (A) Multi‐parameter ranking of the 9 candidate compounds. (B) Synergistic effect of the G–S combination in Panc‐1 cells under “immune‐cold” vs. “immune‐hot” mimicry conditions. (C) Schematic from TME classification of PDAC tissues (*n* = 21) to establishment of a corresponding PDO cohort for *ex vivo* drug validation. (D) Heatmap of immune cell infiltration in consensus‐defined “immune‐hot” (*n* = 4) vs. “immune‐cold” (*n* = 17) PDAC patients. (E,F) Drug response in TME‐stratified PDOs. Bright‐field images (E) and relative viability quantification (F) after 48‐h treatment with single agents or combination. Scale bar: 50 µm. (G) Comparison of IC50 values for the G‐S treatment in “immune‐hot” vs. “immune‐cold” PDOs. (H) Dose‐response curves of the G‐S treatment in “immune‐hot” and “immune‐cold” PDOs. Data were represented as the mean ± SD. Statistical significance was determined by two‐way ANOVA (F) and by the Mann–Whitney U test (G). ^*^, *p* < 0.05; ^***^, *p* < 0.001; ns, not significant. GEM, gemcitabine.

To rank the nine candidate compounds identified from the integrated in vitro and in silico screens, we established a multi‐parameter ranking system to evaluate their potential for subtype‐specific therapy. Each compound was scored according to (i) the IC50 fold change (Cold/Hot‐mimicry) in combination treatment, (ii) the relative cell growth inhibition rate, and (iii) the statistical significance (*p*‐value) of its differential sensitivity between the two immune contexts. This multi‐criteria assessment identified SAHA as the top‐ranking candidate, showing the most promising profile for targeting IE‐PDAC (Figure 2A). We next evaluated the synergistic effect between gemcitabine and the top candidate, SAHA, under the “immune‐hot” condition. A markedly higher synergy score was observed in the “immune‐hot” mimicry condition compared to the “immune‐cold” setting (3.68‐fold increase; Figure 2B). This result confirms a strong, context‐dependent synergistic cytotoxicity, indicating that the drug combination achieves superior efficacy within the targeted IE‐TME.

To validate our findings in a more physiologically relevant model, we established a PDO cohort from 21 treatment‐naïve PDAC patients. These PDOs were paired with transcriptomic profiles of the original tumor tissues. The integrated analytical workflow is outlined in Figure 2C. Briefly, patients were stratified into “immune‐hot” and “immune‐cold” subgroups based on CD8^+^ T cell infiltration levels estimated by three transcriptional deconvolution algorithms (CIBERSORT, MCP‐counter, TIMER) [24, 25, 26] (Figure 2D; Figure S2A–E).

The G‐S combination outperformed single‐agent therapy in both PBMC co‐culture and non‐co‐culture settings, yet its relative inhibitory effect was markedly greater when PBMCs were present (Figure 2E,F; Figure S2F,G). This finding aligns with our cell line data, demonstrating that the synergistic cytotoxicity of this regimen is enhanced by an “immune‐hot” condition. Furthermore, the G‐S combination exhibited significantly lower IC50 values in the “immune‐hot” PDOs than in the “cold” ones (Figure 2G), a trend uniformly reflected in the dose‐response curves (Figure 2H).

### 3.3 Identification of FASN and PARP9 as Key Mediators of SAHA Response in Immune‐Enriched PDAC Through Lipid Metabolic Reprogramming

**FIGURE 3:**
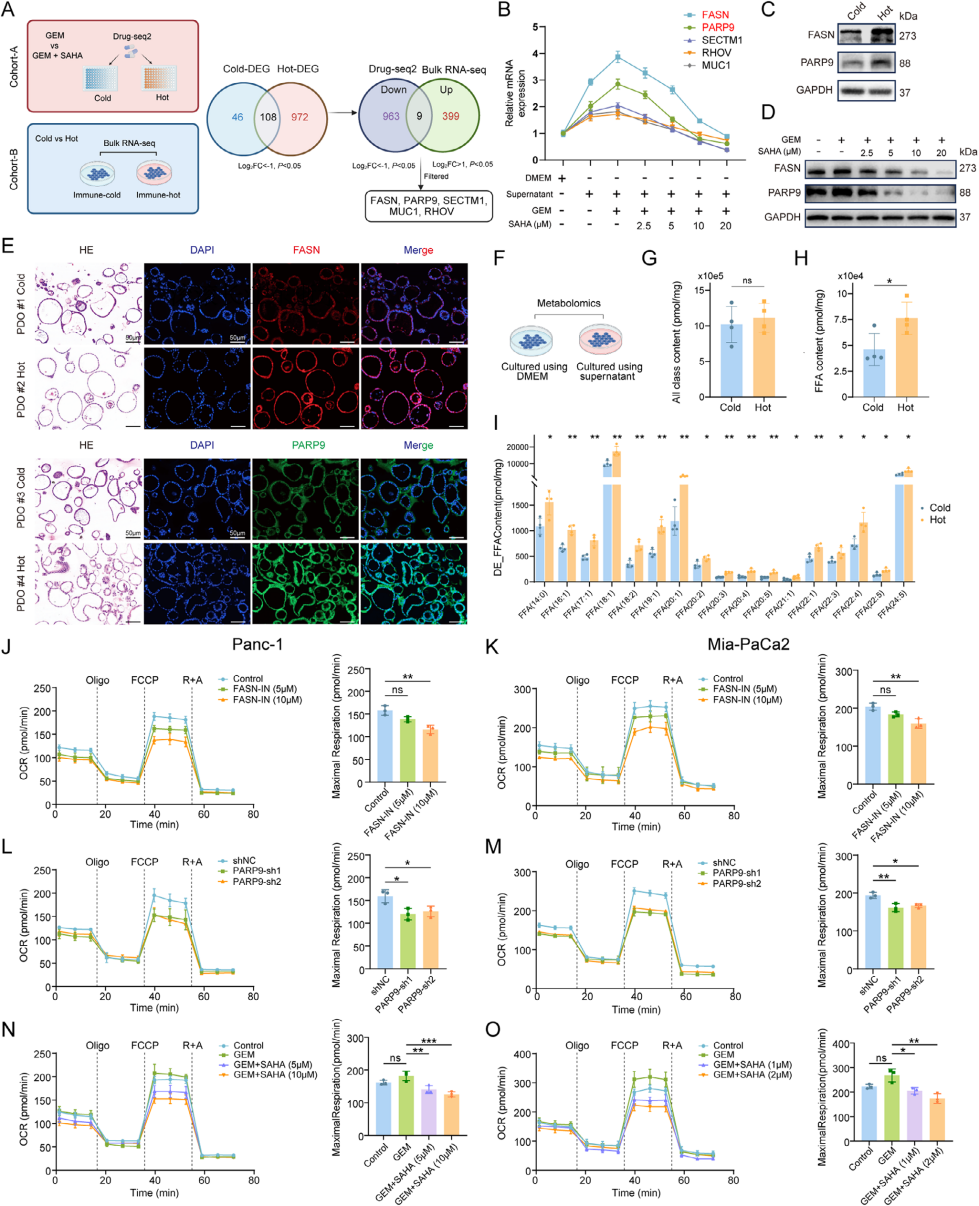
FASN and PARP9 Mediate SAHA Response in Immune‐Enriched PDAC through Lipid Metabolic Reprogramming and OXPHOS Regulation. (A)Identification of key molecules via integration of Drug‐seq2 and Bulk RNA‐seq. (B)qPCR validation of the five candidate genes. (C‐D) Western blot analysis confirming the upregulation of FASN and PARP9 proteins in the “immune‐hot” condition and their dose‐dependent suppression by SAHA in Panc‐1 cells. (E) H&E and IF staining of FASN and PARP9 in stratified PDOs. Scale bar: 50 µm. (F) Graphical strategy for comparative lipidomic analysis of Panc‐1 cells under “immune‐cold” vs. “immune‐hot” mimetic conditions. (G) Comparison of total lipid abundance between “immune‐cold” (*n* = 4) and “immune‐hot” (*n* = 4) conditions. (H) Comparison of total FFA content between “immune‐cold” (*n* = 4) and “immune‐hot” (*n* = 4) conditions. (I) Abundance of differentially expressed FFA between “immune‐cold” (*n* = 4) and “immune‐hot” (*n* = 4) conditions. (J‐K) OCR measurements in Panc‐1 (J) and Mia‐PaCa2 cells (K) treated with FASN inhibitor at 5 and 10 µm. (L,M) OCR measurements in Panc‐1 (L) and Mia‐PaCa2 cells (M) with PARP9‐knockdown. (N,O) OCR measurements in Panc‐1 (N) and Mia‐PaCa2 cells (O) treated with gemcitabine alone or in combination with SAHA. Data were represented as the mean ± SD. Statistical significance was determined by unpaired two‐tailed Student's *t*‐tests (G, H, I) and by one‐way ANOVA with Tukey's post hoc tests (J, K, L, M, N, O). ^*^, *p* < 0.05; ^**^, *p* < 0.01; ^***^, *p* < 0.001; ns, not significant.

We sought to uncover the mediators that sensitize PDAC to the G‐S combination specifically in “immune‐hot” contexts by integrating two complementary transcriptomic approaches: Drug‐Seq2 (Figure S3A,B) and Bulk RNA‐seq (Figure S3C). Using Drug‐Seq2 data from Panc‐1 cells under distinct mimetic conditions, we pinpointed 972 genes specifically downregulated by SAHA in a dose‐dependent manner only in the “hot” condition. Independently, we identified 408 genes that were significantly upregulated in Panc‐1 cells in response to the “immune‐hot” mimetic culture. The convergence of these two gene sets yielded 9 candidates, with five genes—FASN, PARP9, SECTM1, MUC1, and RHOV, emerging as the most statistically significant hits (Figure 3A).

Subsequent qPCR validation of the five candidate genes revealed that FASN and PARP9 displayed two key characteristics: the highest induction under the “immune‐hot” culture and the strongest dose‐dependent suppression by SAHA (Figure 3B), leading to their selection as genes of interest. These two genes were significantly upregulated in PDAC tissues compared with adjacent normal samples (Figure S3D). Western blot analysis further confirmed that FASN and PARP9 were upregulated under immune‐hot‐mimicking conditions and downregulated upon G‐S treatment (Figure 3C,D). Consistently, IF staining showed higher expression of FASN and PARP9 in PDOs established from “immune‐hot” PDAC tissues (Figure 3E). These results thus identified FASN and PARP9 as key players in the IE‐PDAC.

Based on the GSEA that demonstrated significant enrichment of the fatty acid metabolism within the IE signature (Figure S3E), we observed a state of lipid metabolic reprogramming underpinned by this specific TME. To test this, we conducted quantitative lipidomic profiling on Panc‐1 cells cultured under different conditions (Figure 3F; Figure S3F). While total lipid abundance remained unchanged between conditions (Figure 3G), we observed significant alterations in several lipid subclasses in the “immune‐hot” context (Figure S3G). Among these, the free fatty acid (FFA) pool was notably expanded, showing a marked increase in total content (Figure 3H). Further analysis showed that 17 of the 33 detected FFAs were significantly upregulated and none were downregulated, indicating a specific and coordinated enhancement of FFA metabolism in the “immune‐hot” microenvironment (Figure 3I).

Given the identification of FASN and PARP9 as key mediators of therapy response in IE‐PDAC, and the established role of FASN as a rate‐limiting enzyme in fatty acid synthesis [27], we reasoned that both contribute to the characteristic FFA accumulation observed in the “immune‐hot” TME. PARP9, while traditionally studied in DNA damage and interferon signaling, has recently been implicated in lipid metabolism [28]. Therefore, we generated stable PARP9‐knockdown cell lines (Figure S3H,I) and performed quantitative lipidomic profiling (Figure S3J). Strikingly, while total lipid levels remained unchanged, PARP9 knockdown led to a significant reduction in overall FFA content (Figure S3K,L). Among the 28 detected FFAs, 13 were significantly downregulated in PARP9‐deficient cells, and none were upregulated, providing direct evidence that PARP9 is required to maintain the heightened FFA levels in the “immune‐hot” context (Figure S3M).

Given fatty acids are important substrate for oxidative phosphorylation (OXPHOS), we hypothesized that the disturbance of FASN and PARP9 may affect energy production in PDAC cells. We assessed mitochondrial respiration using Seahorse assays and found that either pharmacological inhibition of FASN or genetic knockdown of PARP9 significantly diminished maximal mitochondrial respiration, as reflected by reduced OCR. Pharmacological inhibition of FASN and genetic knockdown of PARP9 each robustly attenuated maximal mitochondrial respiration in Panc‐1 cells (Figure 3J,L), and similarly, the same treatments mirrored this suppressive effect in Mia‐PaCa2 cells (Figure 3K,M). We next examined whether OXPHOS responds to the proposed G‐S combination. In PDAC cells cultured under “immune‐hot” mimetic conditions, gemcitabine monotherapy led to a modest upward trend in OXPHOS levels, whereas the addition of SAHA markedly suppressed OXPHOS in a dose‐dependent manner (Figure 3N,O). Together, these data support a model wherein FASN and PARP9 jointly enhance FFA synthesis, promote OXPHOS, and drive ATP production in PDAC cells, thereby supplying energy under “immune‐hot” conditions. SAHA can abrogate this unexpected OXPHOS activation and curtail the energy supply.

### 3.4 HDAC3 Forms a Transcriptional Complex with FOSL1 and JUNB to Upregulate FASN Expression in Immune‐Enriched PDAC

**FIGURE 4:**
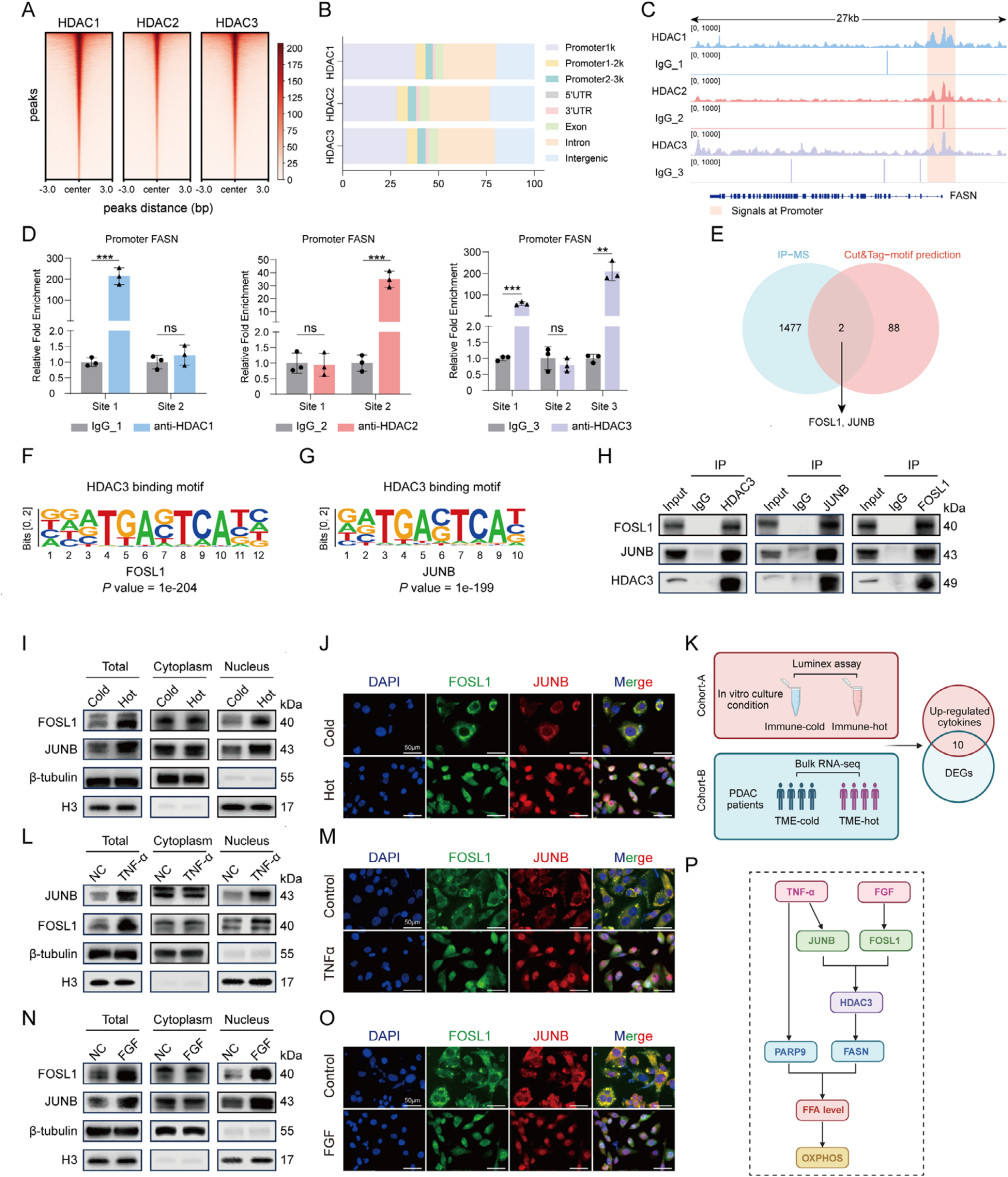
HDAC3 Partners with FOSL1 and JUNB to Form a Transcriptional Complex that Upregulates FASN in Immune‐Enriched PDAC. (A)Distribution of CUT&Tag signal intensities at HDAC1/2/3 binding peaks in Panc‐1 cells under “immune‐hot” mimicry. (B) Stacked bar plots showing genomic annotation of HDAC1/2/3 binding regions. (C) IGV snapshots showing HDAC1/2/3‐binding signals in the TSS region, showing that HDAC1/2/3 are enriched in the *FASN* promoter regions. (D)Validation of HDAC1/2/3 binding to the *FASN* promoter region by CUT&Tag‐qPCR. (E)Intersection of HDAC1/2/3 interactors (IP‐MS) with transcription factors predicted from CUT&Tag motif analysis. (F,G) DNA binding motifs for FOSL1 (F) and JUNB (G) from motif analysis of HDAC3 CUT&Tag peaks. (H) Co‐IP and Western blot validation of the HDAC3‐FOSL1‐JUNB complex in nuclear extracts from Panc‐1 cells cultured under “immune‐hot” conditions. (I,J) Western blot and IF analysis of FOSL1 and JUNB expression and distribution in “immune‐hot” vs. “immune‐cold” conditions. Scale bar: 50 µm. (K) Identification of upstream AP‐1‐activating cytokines via integration of cytokine and transcriptomic datasets. (L,M) Western blot and IF analysis of FOSL1 and JUNB in response to TNF‐α stimulation. Scale bar: 50 µm. (N,O) Western blot and IF analysis of FOSL1 and JUNB in response to FGF stimulation. Scale bar: 50 µm. (P) Schematic depicting how external cytokine signaling affects FASN/PARP9 leading to FFA accumulation and elevated OXPHOS. Data were represented as the mean ± SD. Statistical significance was determined by unpaired two‐tailed Student's *t*‐tests (D). ^**^, *p* < 0.01; ^***^, *p* < 0.001; ns, not significant.

Given that SAHA is a pan‐HDAC inhibitor, we further study how HDAC family members mediate lipid metabolic reprogramming under “immune‐hot” exposure, thereby uncovering the mechanism underlying the superior efficacy of the G‐S combination therapy in “immune‐hot” compared to “immune‐cold” subtypes. We first performed genome‐wide CUT&Tag to identify genes potentially regulated by HDAC1/2/3 (Figure 4A,B; Figure S4A). These findings revealed significant binding of all three HDACs to the promoter region of *FASN* (Figure 4C), suggesting their direct involvement in regulating *FASN* transcription, of which finding that was further validated by CUT&Tag‐qPCR (Figure 4D). However, no evidence was observed for direct binding between HDACs and PARP9.

Motif analysis of HDAC1/2/3 CUT&Tag peaks predicted 88 potential transcription factors (TF) binding sites. To identify which of these TFs physically interact with the HDACs, we performed immunoprecipitation‐mass spectrometry (IP‐MS) using HDAC1/2/3 antibodies under “immune‐hot” mimetic conditions. The overlap between the IP‐MS proteins and the predicted motifs identified two candidates: FOSL1 and JUNB, which are well‐characterized as components of the AP‐1 complex (Figure 4E). Although the motif predictions implicated all three HDACs, the IP‐MS data indicate that direct physical interactions with these two TFs are likely specific to HDAC3. GO functional enrichment analysis of the DNA peaks bound by HDAC3 showed a strong association with cellular metabolic processes (Figure S4B). This finding suggests a potential cooperative mechanism between HDAC3 and AP‐1 in transcriptional regulation, potentially targeting metabolic pathways.

Significant enrichment of FOSL1 and JUNB DNA binding motifs was observed in HDAC3 CUT&Tag peaks (Figure 4F,G). A physical interaction among HDAC3, FOSL1, and JUNB, initially confirmed by Co‐IP in nuclear extracts (Figure 4H). Direct visual evidence of their nuclear co‐localization was observed under “immune‐hot‐mimicry” culture (Figure S4C). Interestingly, we observed a marked nuclear accumulation of the AP‐1 components FOSL1 and JUNB under “immune‐hot” conditions, as evidenced by increased total and nuclear protein levels without significant changes in their cytoplasmic distribution (Figure 4I,J). Concomitantly, HDAC3 exhibited enhanced nuclear translocation in the “immune‐hot” context, as collectively demonstrated by western blot (Figure S4D) of nuclear fractions and IF staining (Figure S4E). Collectively, these findings support a model wherein the IE‐condition upregulates nuclear protein levels of HDAC3, FOSL1, and JUNB, facilitating the assembly of a ternary transcriptional complex that translates immune signals into metabolic reprogramming via the regulation of targets such as *FASN* in PDAC. In addition, transcriptomic data from TCGA‐PAAD further demonstrated positive correlations between FASN and HDAC3, FOSL1, and JUNB, respectively (Figure S4F).

Based on these findings, we sought to identify the key cytokines in the “immune‐hot” milieu that orchestrate the dynamic assembly of the transcriptional complex. Using a systematic approach combining immune‐mimetic and patient transcriptomic data, we identified 10 candidate cytokines driving AP‐1 nuclear accumulation (Figure 4K). Functional screening in Panc‐1 cells treated with each cytokine (10 ng/mL, 48 h) revealed TNF‐α as the most potent inducer of nuclear JUNB upregulation (Figure S4G,H; Figure 4L,M) and FGF as the strongest enhancer of nuclear FOSL1 (Figure 4N,O; Figure S4G,H), identifying these factors as specific microenvironmental triggers of AP‐1 activation. We further observed that TNF‐α treatment for 48 h significantly upregulated total PARP9 protein levels by western blot (Figure S4I), inferring that TNF‐α may potentiate PARP9‐mediated cellular metabolic processes (as illustrated previously). These findings uncover an additional pathway linking immune signaling to metabolic regulation in the PDAC microenvironment.

Overall, we propose a coordinated pathway operating in IE‐PDAC: TNF‐α and FGF upregulate nuclear protein level of JUNB and FOSL1, respectively, assembling into an AP‐1 transcription complex that recruits HDAC3 to cooperatively enhance *FASN* transcription. Meanwhile, TNF‐α strengthens the immune‐metabolic link by upregulating PARP9 level. These mechanisms collectively drive elevated FFA levels, OXPHOS, and energy production, ultimately promoting tumor resilience and immune evasion (Figure 4P). Overall, these findings outline a multi‐faceted signaling network that connects immune context to metabolic reprogramming in PDAC.

### 3.5 The G‐S Combination Therapy Exhibits Superior Anti‐Tumor Efficacy in Immunocompetent Mouse Models

**FIGURE 5:**
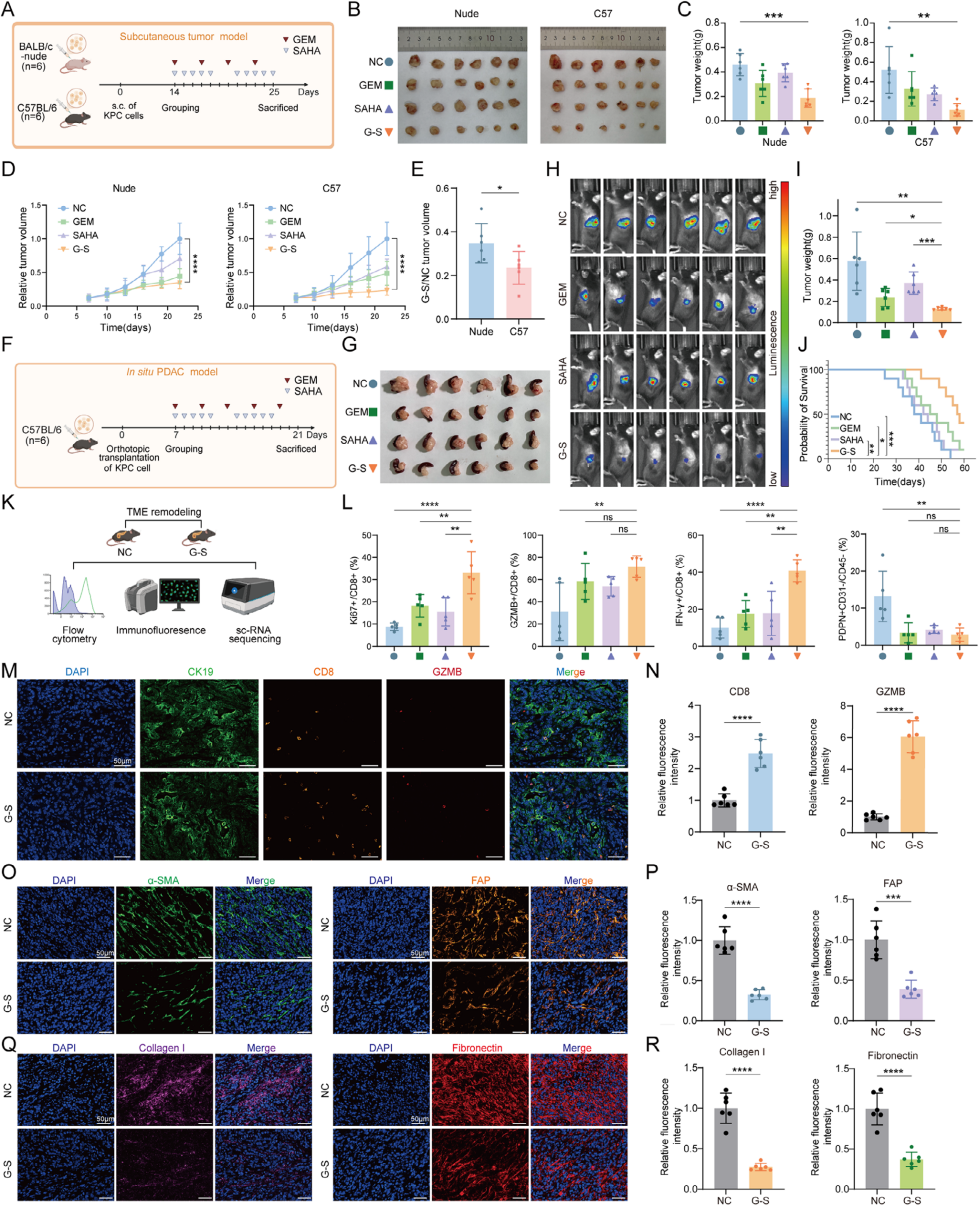
The Gemcitabine–SAHA (G–S) Combination Therapy Shows Enhanced Anti‐Tumor Efficacy by Reprogramming the TME in Immunocompetent PDAC Models. (A) Diagram of the in vivo therapeutic strategy in subcutaneous KPC tumor‐bearing models in immunodeficient and immunocompetent mice. (B‐E) Evaluation of the G‐S combination on tumor growth in BALB/c nude and C57BL/6J mice (*n* = 6 per group). Representative images (B), relative tumor weight (C), relative tumor growth curves (D), and endpoint tumor volume ratio (E) for indicated groups were presented. (F)Diagram of the in vivo therapeutic strategy in orthotopic KPC tumor‐bearing model in C57BL/6J mice. (G–J) Evaluation of the G‐S combination on tumor growth in situ PDAC mice model (*n* = 6 per group). Representative images (G), bioluminescence imaging (H), tumor weight (I), and Kaplan‐Meier curves (J) for indicated groups were presented. (K) Schematic model unraveling the landscape of TME remodeling following G–S treatment. (L) Flow cytometry analysis of tumor‐infiltrating immune cells. Quantification shows the frequencies of activated CD8^+^ T cell subsets (Ki‐67^+^, Granzyme B^+^, and IFN‐γ^+^) and the level of CAF infiltration. (M,N) mIHC validation of enhanced CD8^+^ T cell infiltration and activation. Representative images (M) and quantification of CD8 and Granzyme B‐positive cells (N). Scale bar: 50 µm. (O,P) IF analysis of CAF markers following combination therapy. Representative images (O) and quantification (P) of α‐SMA and FAP. Scale bar: 50 µm. (Q,R) IF analysis of ECM deposition following combination therapy. Representative images (Q) and quantification (R) of Collagen‐I and Fibronectin. Scale bar: 50 µm. Data were represented as the mean ± SD. Statistical significance was determined by one‐way ANOVA with Tukey's post hoc tests (C, D, I, L), by the log‐rank tests (J), and by unpaired two‐tailed Student's t‐tests (E, N, P, R). ^*^, *p* < 0.05; ^**^, *p* < 0.01; ^***^, *p* < 0.001; ^****^, *p* < 0.0001; ns, not significant. G‐S, gemcitabine‐SAHA.

To investigate the therapeutic potential of the G‐S combination in vivo, we established subcutaneous tumor models using KPC cells in both immunodeficient nude mice and immunocompetent C57BL/6 mice (Figure 5A). The G‐S combination provided superior tumor control over monotherapies in both models (Figure 5B–D), with its efficacy being markedly better in immunocompetent mice than in immunodeficient mice, as shown by reductions in final growth measures (Figure 5E).

To further evaluate the drug efficacy within a physiological TME, we established an orthotopic PDAC model in C57BL/6J mice (Figure 5F). Consistently, the G‐S combination achieved significantly greater tumor control than either agent alone, as evidenced by reduced tumor volume (Figure 5G,H) and lower tumor weight (Figure 5I) at the endpoint. Moreover, Kaplan‐Meier survival analysis revealed that the combination therapy significantly prolonged median overall survival compared to all other groups (Figure 5J).

To delineate the TME remodeling following the G‐S treatment, we performed flow cytometry, immunofluorescence, and scRNA‐seq (Figure 5K). Flow cytometry showed the G‐S treatment markedly enhanced the infiltration of activated CD8^+^ T cells compared to either monotherapy, as characterized by significant increases in Ki‐67^+^, granzyme B^+^, and IFN‐γ^+^ subsets. In parallel, the TME exhibited a substantial decrease in CAF infiltration (Figure 5L) after the G‐S treatment, indicating a concerted remodeling of both immune and stromal compartments. These findings were further corroborated at the spatial tissue level by immunofluorescence analysis. Tumors subjected to the G‐S therapy exhibited a significant increase in the density of tumor infiltrating CD8^+^ T cells, displaying a potentiated functional state characterized by elevated expression of Granzyme B (Figure 5M,N). Moreover, we observed that the G‐S therapy led to a marked reduction in CAF activation. This was evidenced by the downregulation of α‐SMA and FAP (Figure 5O,P) and was accompanied by a significant impairment in the production of the core ECM proteins Collagen‐I and Fibronectin (Figure 5Q,R).

**FIGURE 6:**
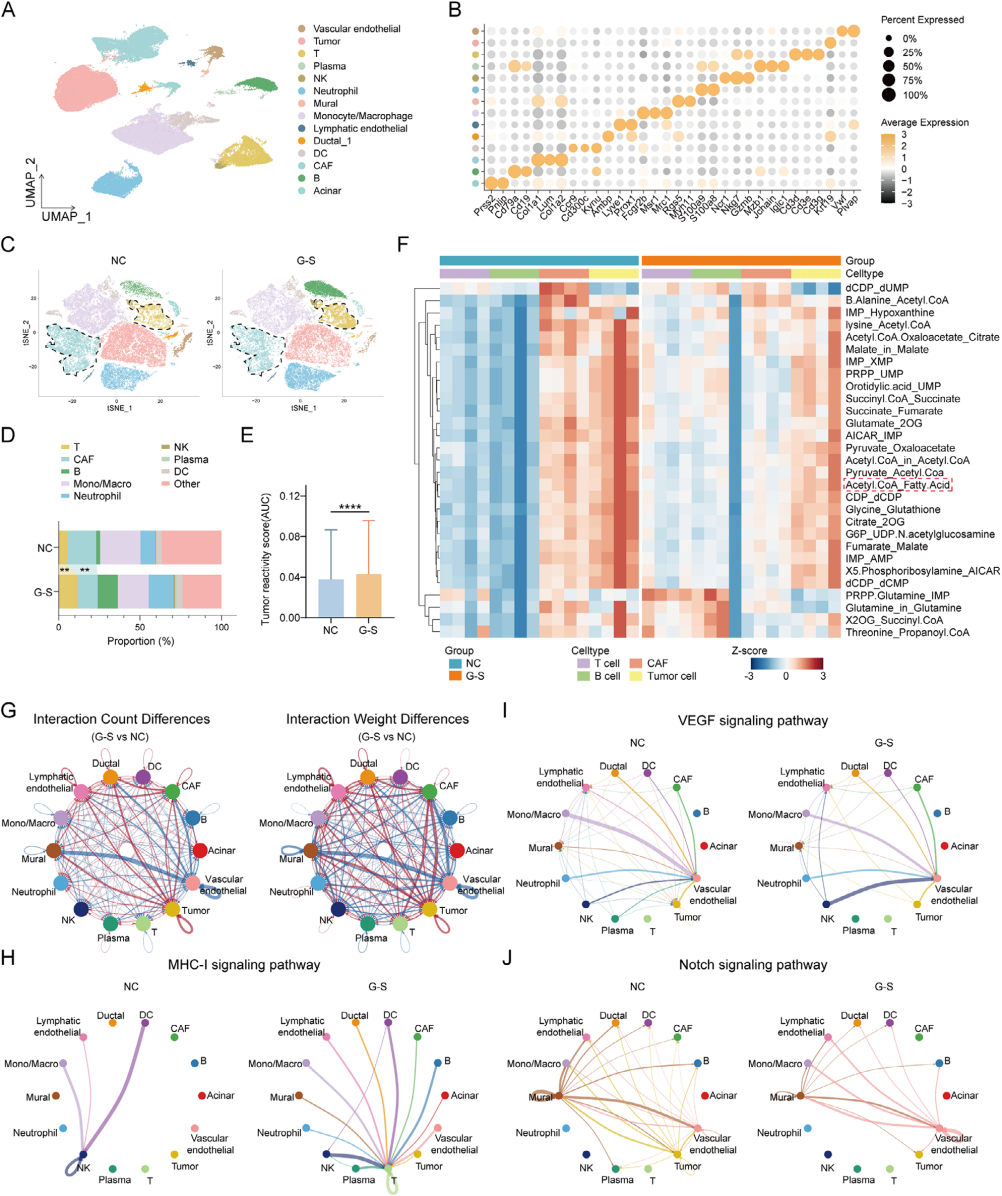
Single‐cell transcriptomic landscape of the orthotopic PDAC TME. (A) UMAP visualization of the TME in the orthotopic PDAC mice model. (B) Bubble heatmap displaying the expression pattern of selected signature genes across 14 major cell clusters. (C) t‐SNE visualization comparing the distribution of major tumor‐infiltrating immune and stromal cell clusters between the NC and G‐S groups (*n* = 4 mice per group). (D) Comparative analysis of major cell cluster proportions between the NC and G‐S groups (*n* = 4 mice per group). (E) Comparison of tumor reactivity scores (AUCell) between the NC and G‐S groups. (F) Single‐Cell Flux Estimation Analysis (scFEA) of differential metabolic fluxes between the NC and G‐S groups across T cells, B cells, tumor cells, and CAFs. (G) Differences in the number (count) and strength (weight) of cell‐cell interactions between the NC and G‐S groups. (H–J) Differential cell‐cell communication in specific signaling pathways between the NC and G‐S groups: MHC‐I signaling network (H), VEGF signaling network (I), and Notch signaling network (J). Data were represented as the mean ± SD. Statistical significance was determined by unpaired two‐tailed Student's *t*‐tests (D,E). ^*^, *p* < 0.05; ^**^, *p* < 0.01; ^****^, *p* < 0.0001. G‐S, gemcitabine‐SAHA.

scRNA‐seq for orthotopic tumor samples identified 14 distinct cell populations based on canonical marker expression (Figure 6A,B). Consistent with the flow cytometry and IF findings, the G‐S group showed a pronounced increase in T cell abundance and a concomitant decrease in CAF proportion compared to controls (Figure 6C,D). Sub‐clustering of major lineages (T cells, B cells, monocytes/macrophages, and CAFs) further verified that the G‐S therapy drives a reprogramming of cellular subset distribution (Figure S5A). Moreover, CAFs exhibit a significantly reduced capacity for stromal deposition following G‐S therapy. In vitro CCK‐8 and colonyformation assays further validated the direct cytotoxic and anti‐proliferative effects of the G‐S therapy targeting human CAFs (Figure S5B,C). In addition, AUCell analysis demonstrated the G‐S treatment significantly elevated the intrinsic anti‐tumor reactivity of tumor cells (Figure 6E).

We next leveraged single‐cell metabolic flux analysis (scFEA) to investigate the divergent metabolic fates following the G‐S therapy (Figure 6F; Figure S5D,E). Specifically, T and B cells shifted toward metabolic programs favoring growth and activation, whereas tumor cells and CAFs exhibited patterns consistent with biosynthetic arrest and energy exhaustion. MEBOCOST analysis, which is a computational approach estimating intercellular metabolic communication [29], further identified the G‐S‐induced alterations in intercellular metabolite abundance (Figure S5F), thereby establishing a functional link between the rewired intracellular metabolism and the reconfigured cell–cell communication network.

Building on these findings, we investigated how the G‐S treatment reshapes cell‐cell communication in the TME. The G‐S administration significantly altered both the quantity and intensity of ligand‐receptor interactions compared to untreated groups (Figure 6G; Figure S6A). Notably, MHC‐I‐mediated signaling pathway was specifically enhanced in the G‐S group, suggesting improved antigen presentation capacity (Figure 6H). Conversely, key pro‐tumorigenic pathways including VEGF and Notch signaling were markedly suppressed in the G‐S group (Figure 6I,J). Additional differential signaling networks are displayed in Figure S6B,C.

To figure out the functional impact of the G‐S treatment, we performed KEGG pathway enrichment analysis on differentially expressed genes. In T cells, cytokine–cytokine receptor interaction was significantly enriched (Figure S6D), indicating that the G‐S treatment modulates cytokine‐mediated crosstalk within the TME. Parallel analysis in tumor cells revealed enrichment of key signaling pathways that may fundamentally alter their malignant behavior or enhance their susceptibility to immune attack (Figure S6E).

To validate the reversal of “metabolic trap” in vivo, we assessed key protein expression in mouse models by IHC. As expected, HDAC3, the direct target of SAHA, was markedly suppressed in the G‐S group compared to controls. The expression of FOSL1 and JUNB, which interact with HDAC3 but are not direct drug targets, remained largely unchanged following G‐S treatment. The expression of the downstream metabolic effectors, FASN and PARP9, was significantly downregulated in the G‐S group (Figure S6F).

Taken together, the G‐S combination therapy creates a restrictive TME by concurrently enhancing immunogenicity, silencing pro‐tumorigenic signaling, and establishing a metabolic landscape that favors immune cells over stromal and malignant components.

### 3.6 SAHA Enhances Anti‐Tumor Efficacy of Chemoimmunotherapy in Humanized Mice and Holds Potential for Improving Outcomes in a High‐Risk Immune‐Enriched Subgroup

**FIGURE 7:**
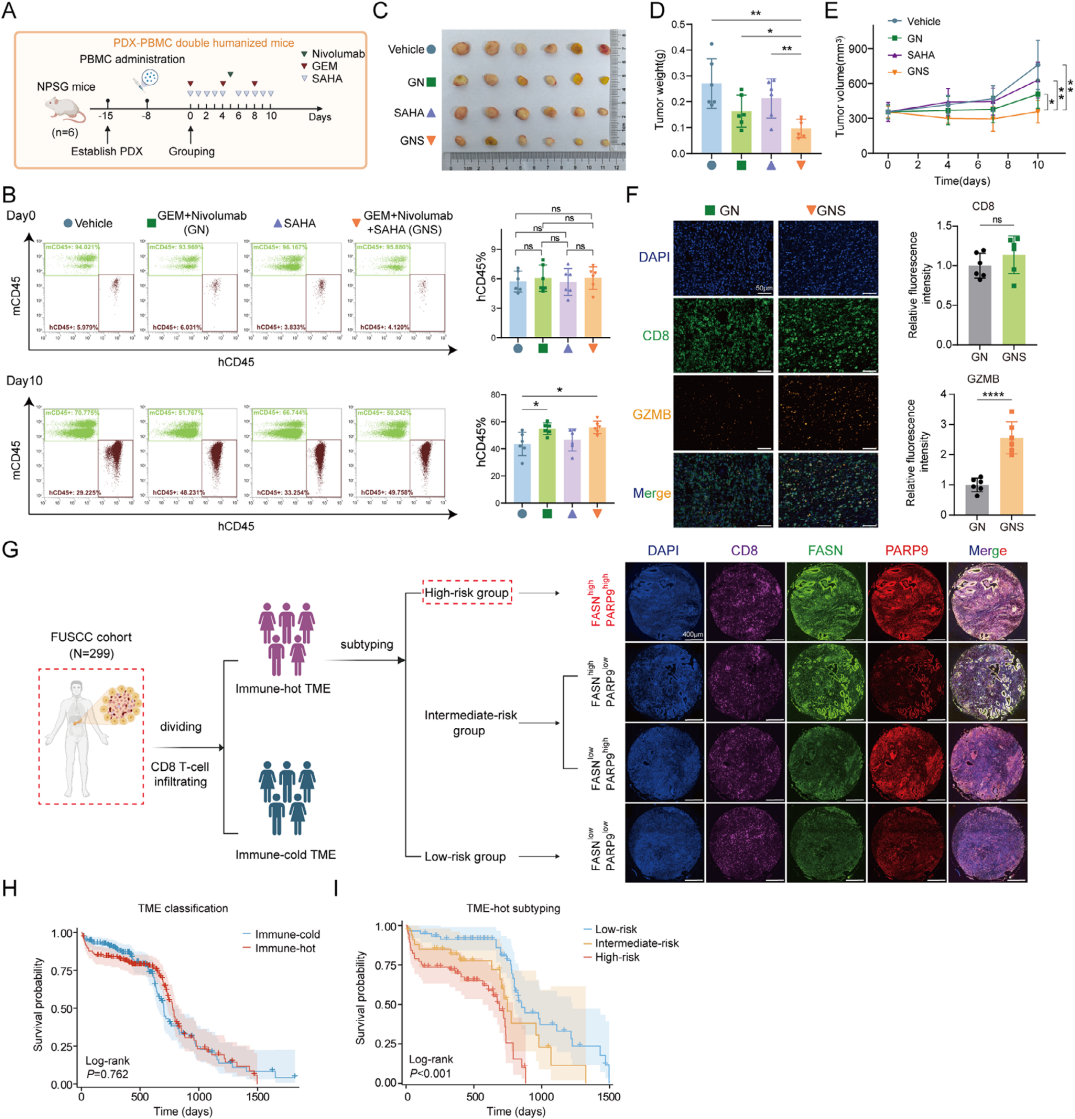
The GNS combination treatment displays potent efficacy in Humanized PDX Mice Model. (A)Diagram of the in vivo therapeutic strategy in humanized PDX model. (B)Flow cytometry analysis of peripheral blood hCD45^+^ at Day 0 and Day 10 after treatment. (C‐E) Evaluation of the GNS combination on tumor growth in humanized PDX model (n = 6 per group). Representative images (C), tumor weight (D), and tumor volume growth curves (E) for indicated groups were presented. (F) Comparative mIHC analysis of CD8^+^ T cell infiltration and GZMB expression between the GN and GNS treatment groups. (G) Representative mIHC images from the FUSCC‐TMA cohort (*n* = 299), showing the stratification of “immune‐hot” PDAC into risk subgroups based on FASN and PARP9 expression. Scale bar: 400 µm. (H) Kaplan–Meier curves of “immune‐hot” vs. “immune‐cold” PDAC patients in the FUSCC cohort. (I) Kaplan–Meier curves of the distinct risk‐subtype of “immune‐hot” group. Data were represented as the mean ± SD. Statistical significance was determined by one‐way ANOVA with Tukey's post hoc tests (B, D, E), by unpaired two‐tailed Student's t‐tests (F), and by log‐rank tests (H,I). ^*^, *p* < 0.05; ^**^, *p* < 0.01; ^****^, *p* < 0.0001; ns, not significant. GN, GEM‐Nivolumab; GNS, GEM‐Nivolumab‐SAHA.

Building on the effect of the G‐S combination on the TME, we next explored whether SAHA could further augment the response to chemoimmunotherapy within an “immune‐hot” TME. To model the humanized TME, we established a dual‐humanized mouse model by engrafting pancreatic cancer PDX tumors into NPSG mice, followed by intravenous injection of human PBMCs (Figure 7A).

Successful engraftment of human CD45^+^ (hCD45^+^) cells in peripheral blood was confirmed one week after PBMC injection. Pre‐treatment hCD45^+^ percentages were comparable, and mice were then randomly assigned to four treatment groups: vehicle, GN, SAHA, and GNS. Following 10 days after treatment, groups receiving GNS exhibited a significant expansion of hCD45^+^ percentage to approximately 50.24% (Figure 7B; Figure S7A).

Evidently, the group receiving GNS therapy exhibited the most potent anti‐tumor effect, as evidenced by tumor sizes (Figure 7C), the lowest final tumor weight (Figure 7D), and tumor growth curves (Figure 7E). Although the proportion of tumor infiltrating CD8^+^ T cells was comparable across groups, their functional state was markedly altered by the addition of SAHA. The GNS regimen significantly enhanced Granzyme B production in CD8^+^ T cells, reflecting a potentiated cytotoxic capacity (Figure 7F). However, in the context of persistent antigen exposure, chronic T‐cell activation often culminates in functional exhaustion [30]. In line with this, PD‐1 has been established as a marker of tumor‐reactive T cells and serves as a critical indicator of responsiveness to immunotherapy [31]. Consistently, we observed that the GNS treatment boosted PD‐1 expression on CD8^+^ T cells (Figure S7B,C), suggesting an enrichment of antigen‐experienced, tumor‐specific T cells. Overall, SAHA does not simply increase T‐cell infiltration but functionally reprograms them, augmenting cytotoxic potential while concurrently inducing a PD‐1‐high state. This SAHA‐driven functional shift likely sensitizes T cells to anti‐PD‐1 checkpoint blockade, thereby underpinning the synergistic efficacy of the triple combination.

**FIGURE 8:**
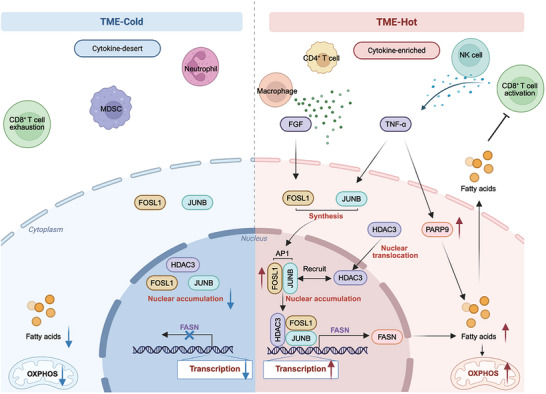
Diagram of the Proposed Model. While immune cells are enriched in the “TME‐hot” PDAC, soluble immune stimulants TNF‐α and FGF upregulate the nuclear protein level of JUNB and FOSL1, respectively, assembling into an AP‐1 transcription complex that recruits HDAC3 to cooperatively enhance *FASN* transcription. Meanwhile, TNF‐α strengthens the immune‐metabolic link by upregulating PARP9 level. These mechanisms collectively drive elevated FFA levels and OXPHOS, inducing chemoresistance of this PDAC subtype. HDAC inhibitor SAHA directly disrupts this FASN/PARP9‐FFA‐OXPHOS axis and indirectly reinvigorates anti‐tumor immunity.

Following the stratification of all patients into “immune‐hot” and “immune‐cold” subgroups by median CD8^+^ T‐cell density, those in the “immune‐hot” group were further stratified based on FASN and PARP9 expression, using the respective medians to define high‐ and low‐ subgroups. With mIHC for 299 samples, three subtype were defined as follows, high‐risk (FASN^high^PARP9^high^), intermediate‐risk (FASN^high^PARP9^low^ or FASN^low^PARP9^high^), and low‐risk (FASN^low^PARP9^low^) subgroups (Figure 7G). While Kaplan‐Meier curves showed comparable survival between the “immune‐hot” and “immune‐cold” groups (Figure 7H), it strikingly revealed that the high‐risk “immune‐hot” subgroup suffered the worst outcome (Figure 7I; Figure S7D,E). We propose this high‐risk subgroup as the patient population most likely to benefit from the triple‐therapy regimen. The mechanism schematic is displayed in Figure 8.

## 4 Discussion

PDAC remains a paramount therapeutic challenge due to its relentless treatment resistance. Conventionally, the IE‐TME confers an intrinsic antitumor advantage associated with improved outcome in many cancers [32, 33, 34, 35, 36], yet this benefit does not extend to IE‐PDAC [7]. This unexpected survival relevance of an IE phenotype is also exemplified in IDH‐mutant astrocytoma, where it is linked to worse prognosis via mechanisms such as GBP1/2‐mediated breach of the blood‐brain barrier and ensuing pro‐tumorigenic crosstalk [10]. Moreover, immunotherapy may remain ineffective for most IE‐PDAC patients, with responses largely confined to less than 1% of patients harboring microsatellite instability‐high tumors [37]. Hence, this superficially friendly IE‐subtype requires more effort to elucidate the unique molecular mechanism responsible for its poor prognosis.

To resolve this, we propose a novel framework: IE‐PDAC is in essence a “metabolic trap”, where the ostensibly favorable immune landscape is hijacked to foster a lipid‐metabolic state that undermines anti‐tumor immunity and drives therapy resistance. SAHA acts as a pivotal breaker of this trap, potentiating chemoimmunotherapy with superior efficacy in IE‐PDAC.

Despite differences in the TME, both “immune‐cold” and “immune‐hot” PDAC still receive identical treatment strategy, majorly chemotherapy. A critical feature of IE‐PDAC is the abundance of soluble immune mediators, like IFN‐γ, FGF, and TNF‐α, which can reprogram tumor cell transcription and alter their drug sensitivity [38]. Beyond these local effects, such cytokines may also fuel a pro‐tumorigenic systemic inflammation state, reflected in elevated peripheral markers like the neutrophil‐to‐lymphocyte ratio (NLR), which is associated with tumor progression and metastasis [39, 40, 41]. However, conventional drug screens, conducted in immune‐devoid systems, fail to capture this complexity. To address this, we established an in vitro “immune‐hot‐mimicry” platform enriched with key immunomodulators to perform high‐throughput drug screening. Multi‐omics analyses confirmed that this culture system established an immunomodulator‐rich milieu, thereby inducing transcriptional remodeling in cancer cells. This platform enabled the discovery of a promising drug combination, subsequently validated in PDO‐PBMC co‐cultures and humanized PDX models.

Mechanistically, we demonstrate how FGF and TNF‐α synergistically establish a “metabolic trap” in IE‐PDAC: both cytokines activate the FOSL1/JUNB/HDAC3 transcriptional complex to upregulate FASN, while TNF‐α independently elevates PARP9 expression. FASN [27, 42] and PARP9 [28] collectively drive FFA biosynthesis, thereby creating a “metabolic trap” in IE‐PDAC. Such lipid‐metabolic reprogramming emerges as a hallmark of the IE‐phenotype. This conclusion supports and extends our prior observations that neoadjuvant chemotherapy can drive PDAC toward an “immune‐hot” state alongside elevated lipid metabolism [12], and that lipid metabolism plays a broad role in immune activation [43, 44].

However, within the IE‐TME, this lipid‐metabolic preference may exert a detrimental effect on antitumor immunity [15]. Enhanced FFA metabolism not only provides tumor cells with a dense energy source via OXPHOS to fuel survival and resistance [45] but also generates a wealth of immunosuppressive species that can directly inhibit T‐cell function [46, 47]. Apart from this, these upstream cytokines themselves can directly subvert immunity. As reported, TNF‐α acts as a key mediator of necrosis‐induced inflammation [48, 49]. Unlike immunogenic cell death pathways of necroptosis and pyroptosis, necrosis‐driven inflammation could attract immune cells yet foster an immunosuppressive milieu that ultimately impairs antitumor immunity [50, 51]. Similarly, FGF signaling has been implicated in fostering immunosuppression [52]. Thus, rather than supporting effective immunity, cytokines such as FGF and TNF‐α represent the “detrimental arm” of the IE‐PDAC, orchestrating a lipid‐rich environment that corrupts the immune landscape and contributes to poor prognosis.

Combining in vitro high‐throughput screening with in silico prediction, we identified the pan‐HDAC inhibitor SAHA as a promising sensitizer for PDAC chemoimmunotherapy. Previous studies have largely focused on its canonical epigenetic mechanisms, which drive direct tumor cytotoxicity through tumor suppressor reactivation (e.g., INK4 family) [53] and suppression of protumorigenic pathways [54]. Emerging evidence further indicates that SAHA can reshape the pancreatic TME toward an immunepermissive state by enhancing tumor infiltrating T‐cell recruitment [55]. Preclinical studies support SAHA's efficacy and tolerability both as monotherapy and in combination regimens [56, 57, 58], underscoring its translational potential for PDAC. However, its specific role within the IE‐PDAC subtype remains poorly understood.

In this study, we demonstrate that SAHA disrupts the “metabolic trap” characteristic of IE‐PDAC via dual inhibition of FASN and PARP9, thereby suppressing cytokine‐driven FFA accumulation. Our in vivo single‐cell sequencing data demonstrated that the G‐S regimen exerts a dual mechanism: First, it blocks tumor cell lipogenesis and redirects metabolic influx in a cell‐type‐specific manner. This reprogramming promotes T‐cell activation while inducing CAF exhaustion. Second, independent of metabolic effects, G‐S therapy holds significant potential for directly killing both tumor cells and CAFs. Furthermore, evaluation in a humanized PDX model confirmed the superior efficacy of the GNS triple therapy over chemoimmunotherapy (GN) alone, supporting its clinical feasibility for the IE‐PDAC subtype. Thus, SAHA not only recruits more soldiers to battle but empowers the existing soldiers to fight more effectively.

The present study has several limitations to disclose. First, our findings elucidate the therapy‐sensitizing mechanism of SAHA intrinsic to tumor cells but are confined to this intracellular context, leaving its direct immunomodulatory effects on T cells unexplored. Second, although humanized PDX models possess human immune components, they may not fully replicate the complexity, spatial organization, or functional state of the original patient's tumor immune microenvironment. The finite lifespan also limits their utility for long‐term studies, especially for modeling certain clinical scenarios like dose reduction or treatment suspension. Finally, the lack of clinical validation in patient cohorts remains a gap that will be the focus of further investigation. To address this gap and expedite clinical translation, we will next conduct a biomarker‐guided Phase I trial in patients with resected PDAC stratified by IE‐subtype signatures, assessing the safety and preliminary efficacy of adjuvant GNS therapy.

Collectively, our findings delineate an epigenetic‐metabolic‐immune axis existing in IE‐PDAC, which SAHA effectively disrupts to exert its therapeutic effect, offering the rationale for prioritizing SAHA‐based combinations in the subset of immune‐enriched, FASN^high^/PARP9^high^ PDAC patients.

## Author Contributions

C.C., D.L., Y.L., and Z.W. performed experiments. C.C. and D.L. conceived the study, processed raw sequencing data, and performed all bioinformatics analysis. C.Z., Z.Z., L.J., and Y.C. supervised the sample collection and performed the pathological examination. J.C. and J.X. participated in discussions. C.C. and M.W. proofread the manuscript. R.T., X.Y., and S.S. supervised the study. All of the authors have read and approved the paper.

## Conflicts of Interest

The authors declare no conflicts of interest.

## Supporting information




**Supporting File**: advs74904‐sup‐0001‐SuppMat.pdf.

## Data Availability

Single‐cell RNA sequencing data from 41 PDAC patients were obtained from the Gene Expression Omnibus (GEO: https://www.ncbi.nlm.nih.gov/geo, GSE155698) [59] and Genome Sequence Archive (GSA: https://bigd.big.ac.cn/gsa, CRA001160) [60]. The mRNA expression data for FASN and PARP in normal pancreatic tissues from the Genotype‐Tissue Expression (GTEx) project and in pancreatic cancer tissues from The Cancer Genome Atlas (TCGA) project were retrieved from UCSC Xena (http://xena.ucsc.edu/). The sequencing data of scRNA‐seq was deposited in the National Omics Data Encyclopedia (https://www.biosino.org/node/, no. OEZ00021847).
