# Supplementary material for: Vorinostat Potentiates Chemoimmunotherapy in Immune‐Enriched Pancreatic Cancer
Source: Adv Sci (Weinh). 2026 Apr 13;13(35):e21844. doi: 10.1002/advs.202521844 (PMC13292265; doi:10.1002/advs.202521844)
Supplement: Supplementary file 1 — Supporting File: advs74904‐sup‐0001‐SuppMat.pdf. [file ADVS-13-e21844-s001.pdf]

Supplemental Figures

Figure S1. Supplemental Data to Figure 1 on Integrated Drug Screening and TME Subtype Characterization.

(A) Determination of optimal co-culture ratio. Levels of IFN-γ and TNF-α in supernatants from PBMCs co-cultured with Panc-1 cells at indicated ratios were measured by ELISA.

(B-D) Proteomic validation of immune activation in co-culture conditioned medium. PCA plot (B) of protein abundance profiles shows clear separation between PBMC/ Panc-1 co-culture (5:1) and Panc-1 monoculture supernatants. Volcano plot (C) of differentially expressed proteins. KEGG pathway enrichment analysis (D) of differentially expressed proteins.

(E) The percentage of different cell clusters in 41 PDAC samples.

(F) Stratification of PDAC samples based on immune cell abundance. Heatmap displays the relative abundance of major immune cell types in immune-high versus immune-low groups, as determined by scRNA-seq profiling.

(G) Stratification of PDAC samples based on T cell abundance. Bar plots compare the average percentage of T cells between T cell-high and T cell-low groups defined by scRNA-seq profiling.

(H, I) Analysis by the OncoPredict algorithm shows the predicted IC50 values for the 9 compounds across distinct TME classifications, showcasing stratification based on overall immune-cell infiltration (H) and specific T-cell abundance (I).

Data were represented as the mean ± SD. Statistical significance was determined by unpaired two-tailed Student's t-tests (G) and by Mann-Whitney U tests (H, I).

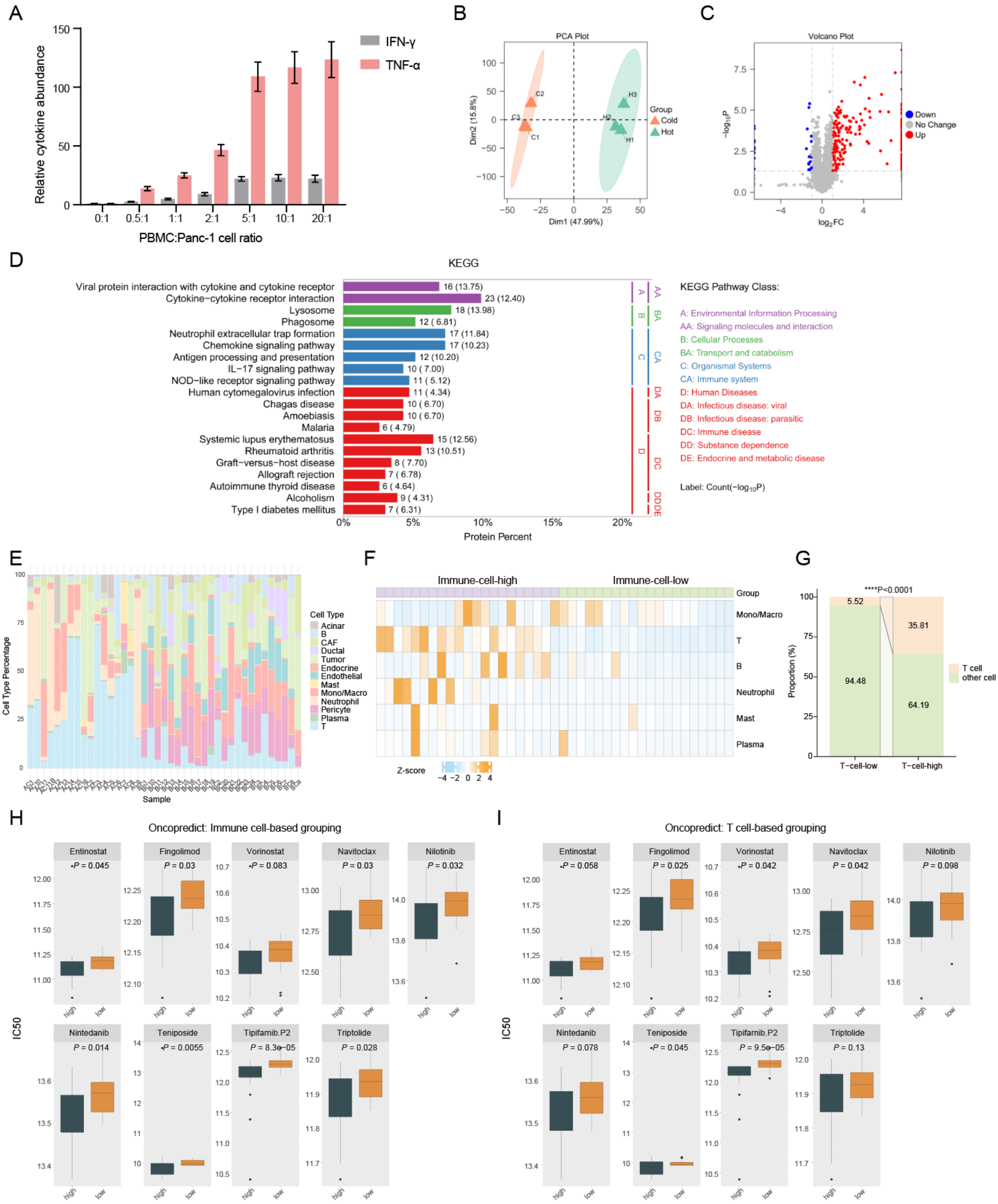
A
Relative cytokine abundance
IFN-γ
TNF-α
PBMC:Panc-1 cell ratio
0:1
0.5:1
1:1
2:1
5:1
10:1
20:1
B
PCA Plot
Dim1 (47.99%)
Dim2 (15.8%)
Group
Cold
Hot
C
Volcano Plot
log2FC
−log10P
Down
No Change
Up
D
KEGG
Viral protein interaction with cytokine and cytokine receptor 16 (13.75)
Cytokine−cytokine receptor interaction 23 (12.40)
Lysosome 18 (13.98)
Phagosome 12 (6.81)
Neutrophil extracellular trap formation 17 (11.84)
Chemokine signaling pathway 17 (10.23)
Antigen processing and presentation 12 (10.20)
IL−17 signaling pathway 10 (7.00)
NOD−like receptor signaling pathway 11 (5.12)
Human cytomegalovirus infection 11 (4.34)
Chagas disease 10 (6.70)
Amoebiasis 10 (6.70)
Malaria 6 (4.79)
Systemic lupus erythematosus 15 (12.56)
Rheumatoid arthritis 13 (10.51)
Graft−versus−host disease 8 (7.70)
Allograft rejection 7 (6.78)
Autoimmune thyroid disease 6 (4.64)
Alcoholism 9 (4.31)
Type I diabetes mellitus 7 (6.31)
Protein Percent
KEGG Pathway Class:
A: Environmental Information Processing
AA: Signaling molecules and interaction
B: Cellular Processes
BA: Transport and catabolism
C: Organismal Systems
CA: Immune system
D: Human Diseases
DA: Infectious disease: viral
DB: Infectious disease: parasitic
DC: Immune disease
DD: Substance dependence
DE: Endocrine and metabolic disease
Label: Count(−log10P)
E
Cell Type Percentage
Sample
Cell Type
Acinar
B
CAF
Ductal
Tumor
Endocrine
Endothelial
Mast
Mono/Macro
Neutrophil
Pericyte
Plasma
T
F
Immune-cell-high
Immune-cell-low
Group
Mono/Macro
T
B
Neutrophil
Mast
Plasma
Z-score
G
****P<0.0001
Proportion (%)
5.52
94.48
35.81
64.19
T cell
other cell
T−cell−low
T−cell−high
H
Oncopredict: Immune cell-based grouping
IC50
Entinostat P = 0.045
Fingolimod P = 0.03
Vorinostat P = 0.083
Navitoclax P = 0.03
Nilotinib P = 0.032
Nintedanib P = 0.014
Teniposide P = 0.0055
Tipifarnib.P2 P = 8.3e−05
Triptolide P = 0.028
high
low
I
Oncopredict: T cell-based grouping
IC50
Entinostat P = 0.058
Fingolimod P = 0.025
Vorinostat P = 0.042
Navitoclax P = 0.042
Nilotinib P = 0.098
Nintedanib P = 0.078
Teniposide P = 0.045
Tipifarnib.P2 P = 9.5e−05
Triptolide P = 0.13
high
low

Figure S2. Supplemental Data to Figure 2 on Validation of TME Stratification and PDO Drug Response

(A-C) Patient stratification into “immune-hot” and “immune-cold” subgroups using three distinct transcriptional deconvolution algorithms, including CIBERSORT-based classification (A), MCPcounter-based classification (B), TIMER-based classification (C).

(D) Venn diagram shows the overlap of patients classified as “immune-hot” by all three algorithms (CIBERSORT, MCP-counter, and TIMER).

(E) Average $CD8^+$ T cell infiltration levels across distinct TME subtypes.

(F, G) Representative bright-field images and PDO relative viability quantification confirming the dependency on “immune-hot” microenvironment for drug efficacy.

Data were represented as the mean ± SD. Statistical significance was determined by unpaired two-tailed Student’s t-tests (E-G). **, p <0.01. GEM, gemcitabine.

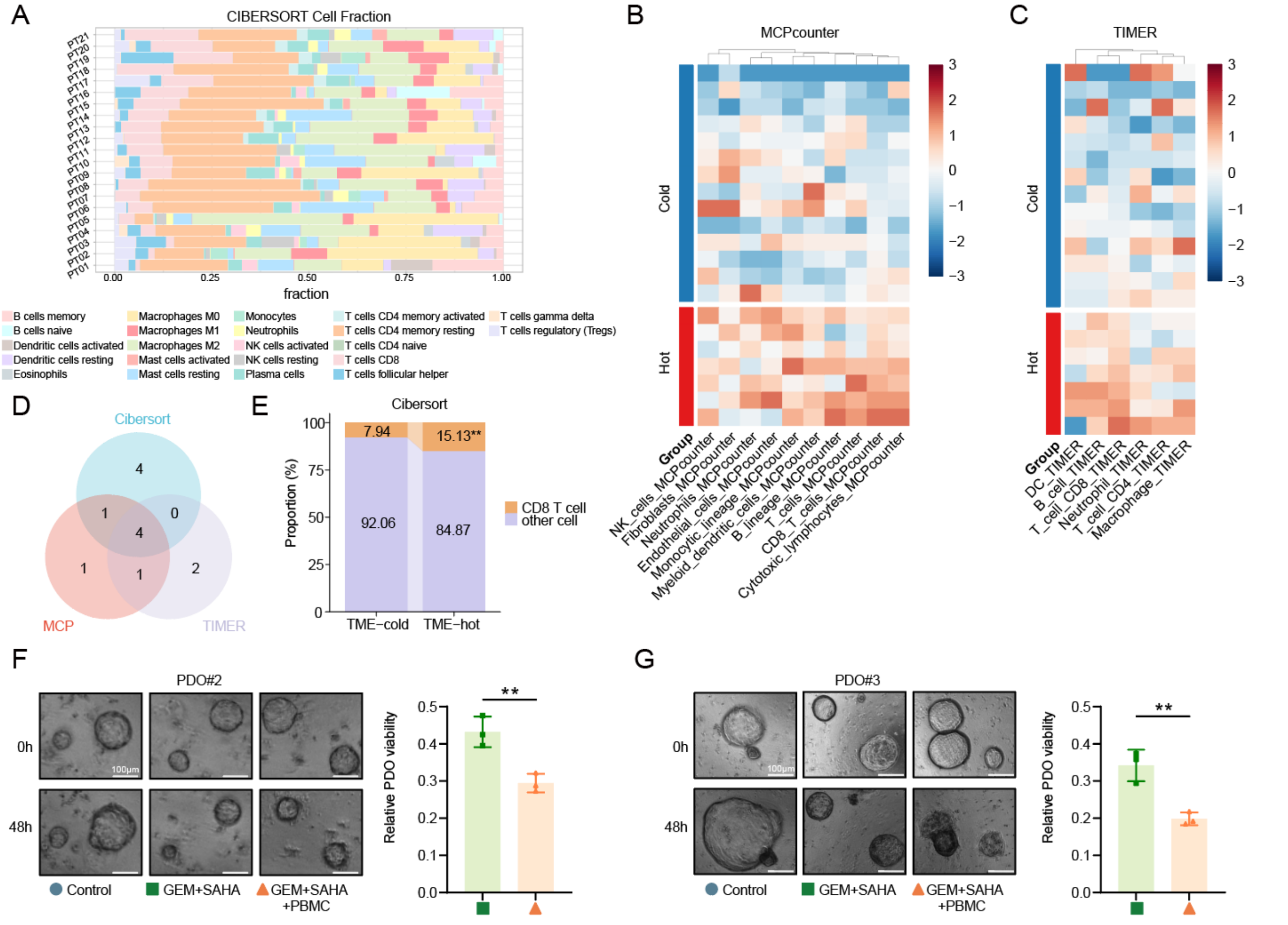

Figure S3. Supplemental Data to Figure 3 on Mechanism Investigation and Lipid Metabolic Reprogramming

(A, B) PCA plot of Drug-Seq2 data from Panc-1 cells under “immune-cold” condition (A) and “immune-hot” condition (B).

(C) Volcano plot displays differentially expressed genes in Panc-1 cells under “immune-hot” versus “immune-cold” mimetic conditions from bulk RNA-seq analysis.

(D) The differential expression of FASN and PARP9 in PDAC tumors compared to normal tissues. Data from TCGA-GTEX PAAD datasets.

(E) GSEA reveals significant enrichment of fatty acid metabolism pathway in immune-enriched transcriptomic signature.

(F) OPLS-DA score plot of quantitative lipidomic profiles shows clear separation between “immune-cold” and “immune-hot” mimetic conditions in Panc-1 cells.

(G) Heatmap of the relative abundance of major lipid subclasses in “immune-cold” versus “immune-hot” conditions.

(H, I) Validation of PARP9 knockdown efficiency in PDAC cell lines. Western blot (left) and qPCR (right) analysis confirm successful PARP9 knockdown in Panc-1 cells (H). Western blot (left) and qPCR (right) analysis confirm successful PARP9 knockdown in Mia-PaCa2 cells (I).

(J) Schematic of the comparative lipidomic analysis in control versus PARP9-knockdown Panc-1 cells.

(K) Comparison of total lipid abundance between control and PARP9-knockdown Panc-1 cells ($n = 4$).

(L) Comparison of total FFA content between control and PARP9-knockdown Panc-1 cells ($n = 4$).

(M) Abundance of differentially expressed FFA species between control and PARP9-knockdown Panc-1 cells ($n = 4$).

Data were represented as the mean ± SD. Statistical significance was determined by unpaired two-tailed Student’s t-tests (D, K-M) and by one-way ANOVA with Tukey’s post hoc tests (H, I). *, $p < 0.05$; **, $p < 0.01$; ***, $p < 0.001$; ns, not significant.

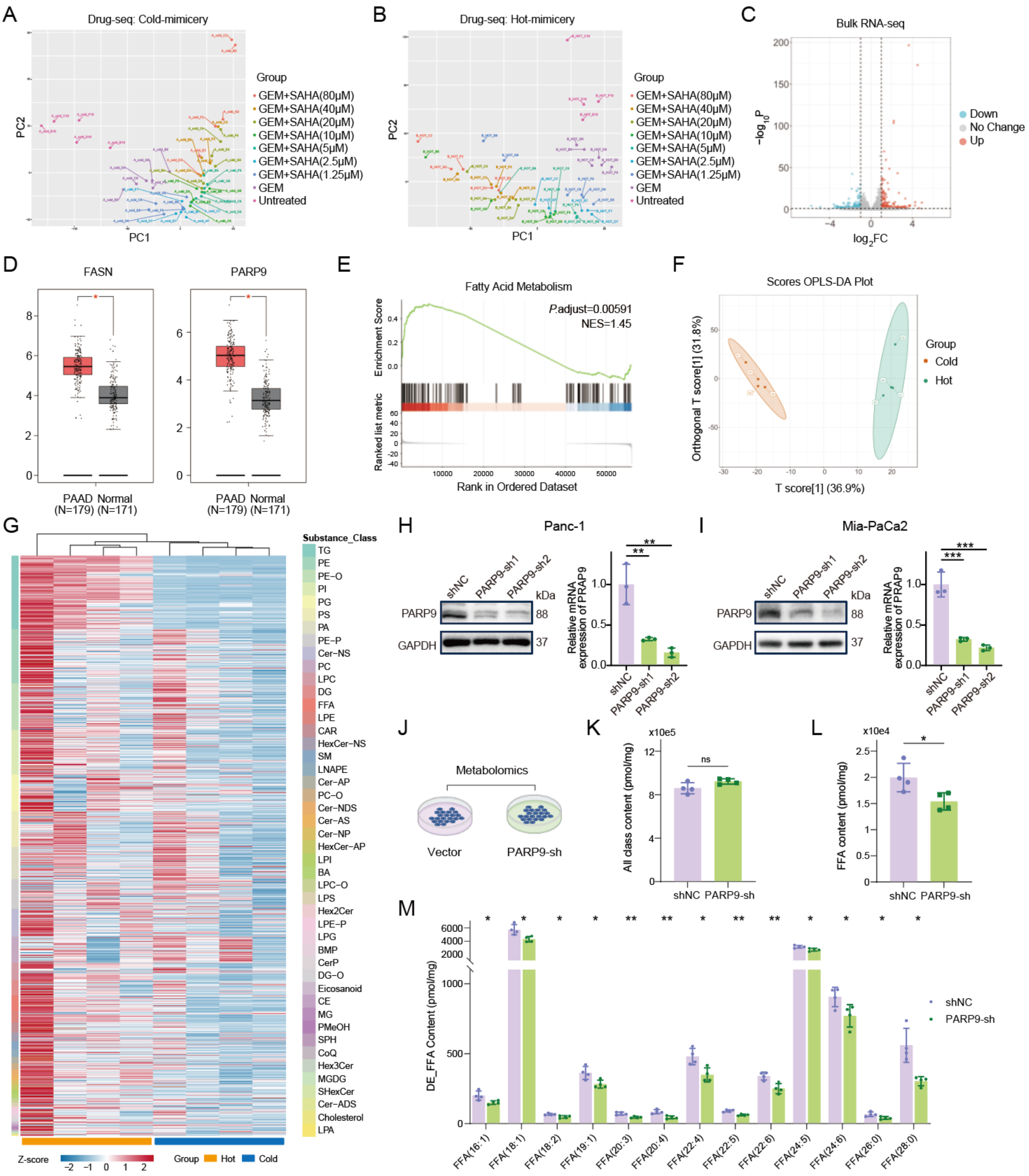

A
Drug-seq: Cold-mimicry
B
Drug-seq: Hot-mimicry
PC1
PC2
Group
GEM+SAHA(80μM)
GEM+SAHA(40μM)
GEM+SAHA(20μM)
GEM+SAHA(10μM)
GEM+SAHA(5μM)
GEM+SAHA(2.5μM)
GEM+SAHA(1.25μM)
GEM
Untreated
C
Bulk RNA-seq
Down
No Change
Up
D
FASN
PARP9
PAAD Normal
(N=179) (N=171)
E
Fatty Acid Metabolism
P.adjust=0.00591
NES=1.45
Enrichment Score
Ranked list metric
Rank in Ordered Dataset
F
Scores OPLS-DA Plot
Group
Cold
Hot
Orthogonal T score[1] (31.8%)
T score[1] (36.9%)
G
Substance_Class
TG
PE
PE−O
PI
PG
PS
PA
PE−P
Cer−NS
PC
LPC
DG
FFA
LPE
CAR
HexCer−NS
SM
LNAPE
Cer−AP
PC−O
Cer−NDS
Cer−AS
Cer−NP
HexCer−AP
LPI
BA
LPC−O
LPS
Hex2Cer
LPE−P
LPG
BMP
CerP
DG−O
Eicosanoid
CE
MG
PMeOH
SPH
CoQ
Hex3Cer
MGDG
SHexCer
Cer−ADS
Cholesterol
LPA
Z-score
Group
Hot
Cold
H
Panc-1
I
Mia-PaCa2
shNC
PARP9-sh1
PARP9-sh2
kDa
PARP9
GAPDH
88
37
Relative mRNA expression of PRAP9
J
Metabolomics
Vector
PARP9-sh
K
x10e5
All class content (pmol/mg)
ns
L
x10e4
FFA content (pmol/mg)
shNC
PARP9-sh
M
DE_FFA Content (pmol/mg)
FFA(16:1)
FFA(18:1)
FFA(18:2)
FFA(19:1)
FFA(20:3)
FFA(20:4)
FFA(22:4)
FFA(22:5)
FFA(22:6)
FFA(24:5)
FFA(24:6)
FFA(26:0)
FFA(28:0)
shNC
PARP9-sh

Figure S4. Supplemental Data to Figure 4 on Mechanistic Insights into HDAC3 Function and Associated Signaling

(A) Average profile of sequencing read distribution across all genes from Cut&Tag data, representing the average signal across all samples.

(B) Gene Ontology (GO) biological process enrichment analysis of genomic regions bound by HDAC3.

(C) Representative IF images demonstrating nuclear co-localization of HDAC3, FOSL1, and JUNB. Scale bar: 50 μm.

(D, E) Western blot and IF analysis of HDAC3 nuclear translocation. Scale bar: 50 μm.

(F) Correlation analysis of gene expression (TCGA-PAAD) for the following pairs: FOSL1 & JUNB; HDAC3 & FASN; FOSL1 & FASN; JUNB & FASN.

(G, H) Western blot of nuclear FOSL1 and JUNB protein expression following treatment with 10 cytokines.

(I) Western blot showing changes in PARP9 protein expression following TNF-α treatment.

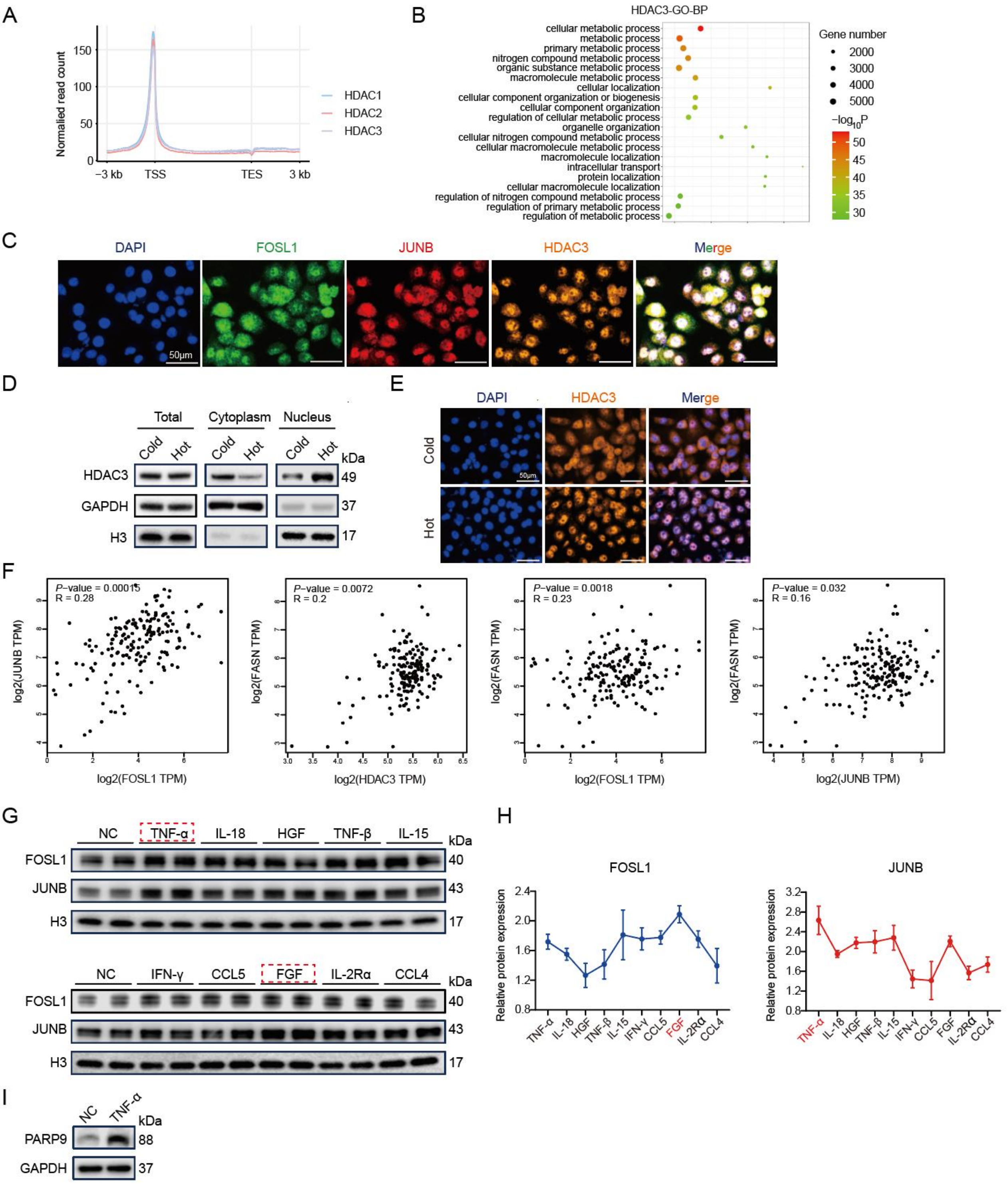
A
Normalied read count
HDAC1
HDAC2
HDAC3
−3 kb
TSS
TES
3 kb
B
HDAC3-GO-BP
cellular metabolic process
metabolic process
primary metabolic process
nitrogen compound metabolic process
organic substance metabolic process
macromolecule metabolic process
cellular localization
cellular component organization or biogenesis
cellular component organization
regulation of cellular metabolic process
organelle organization
cellular nitrogen compound metabolic process
cellular macromolecule metabolic process
macromolecule localization
intracellular transport
protein localization
cellular macromolecule localization
regulation of nitrogen compound metabolic process
regulation of primary metabolic process
regulation of metabolic process
Gene number
2000
3000
4000
5000
−log10P
C
DAPI
FOSL1
JUNB
HDAC3
Merge
50μm
D
Total
Cytoplasm
Nucleus
Cold
Hot
kDa
HDAC3
GAPDH
H3
49
37
17
E
DAPI
HDAC3
Merge
Cold
Hot
F
P−value = 0.00015
R = 0.28
log2(JUNB TPM)
log2(FOSL1 TPM)
P−value = 0.0072
R = 0.2
log2(FASN TPM)
log2(HDAC3 TPM)
P−value = 0.0018
R = 0.23
log2(FASN TPM)
log2(FOSL1 TPM)
P−value = 0.032
R = 0.16
log2(FASN TPM)
log2(JUNB TPM)
G
NC
TNF-α
IL-18
HGF
TNF-β
IL-15
kDa
FOSL1
JUNB
H3
40
43
17
NC
IFN-γ
CCL5
FGF
IL-2Rα
CCL4
H
FOSL1
JUNB
Relative protein expression
I
NC
TNF-α
kDa
PARP9
GAPDH
88
37

Figure S5. Supplemental Data to Figure 6 on Single-Cell Resolution of Metabolic Alterations in the TME following the G-S Therapy

(A) UMAP plots showing the sub-clustering of cellular components and cell percentage within the TME: T cells, B cells, monocytes/macrophages, and CAFs.

(B) Time-course analysis of CAF viability under different treatments.

(C) Colony formation assay of CAF under different treatments.

(D) ScFEA analysis of top 10 upregulated differential metabolic pathways in T cells from the G-S group compared to the NC group.

(E) ScFEA analysis of top 10 downregulated differential metabolic pathways in CAFs from the G-S group compared to the NC group.

(F) Cell-cell communication analysis of differential metabolites based on MEBOCOST. The plot depicts specific metabolite interactions across different cell-cell pairs. Color intensity represents the Log_2 fold change in metabolite abundance, with red indicating higher levels in the G-S group.

Data were represented as the mean ± SD. Statistical significance was determined by one-way ANOVA with Tukey's post hoc test (B) and by unpaired two-tailed Student's t-tests (D, E). *, $p < 0.05$; **, $p < 0.01$; ***, $p < 0.001$; ****, $p < 0.0001$. G-S, gemcitabine-SAHA.

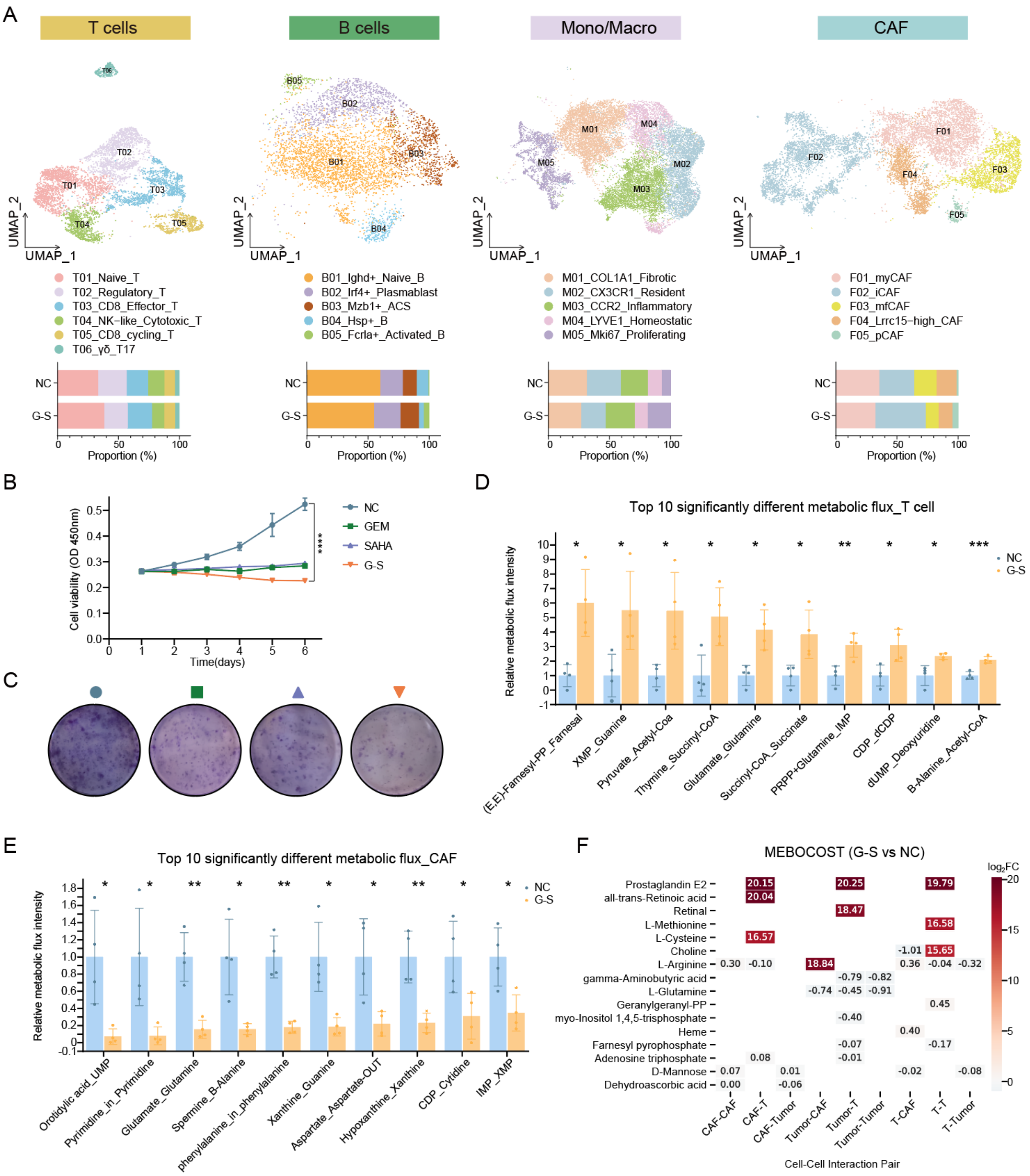
A
T cells
B cells
Mono/Macro
CAF
UMAP_1
UMAP_2
T01_Naive_T
T02_Regulatory_T
T03_CD8_Effector_T
T04_NK−like_Cytotoxic_T
T05_CD8_cycling_T
T06_γδ_T17
B01_Ighd+_Naive_B
B02_Irf4+_Plasmablast
B03_Mzb1+_ACS
B04_Hsp+_B
B05_Fcrla+_Activated_B
M01_COL1A1_Fibrotic
M02_CX3CR1_Resident
M03_CCR2_Inflammatory
M04_LYVE1_Homeostatic
M05_Mki67_Proliferating
F01_myCAF
F02_iCAF
F03_mfCAF
F04_Lrrc15−high_CAF
F05_pCAF
NC
G-S
Proportion (%)
B
Cell viability (OD 450nm)
Time(days)
****
NC
GEM
SAHA
G-S
C
D
Top 10 significantly different metabolic flux_T cell
Relative metabolic flux intensity
(E,E)-Farnesyl-PP_Farnesal
XMP_Guanine
Pyruvate_Acetyl-Coa
Thymine_Succinyl-CoA
Glutamate_Glutamine
Succinyl-CoA_Succinate
PRPP+Glutamine_IMP
CDP_dCDP
dUMP_Deoxyuridine
B-Alanine_Acetyl-CoA
E
Top 10 significantly different metabolic flux_CAF
Relative metabolic flux intensity
Orotidylic acid_UMP
Pyrimidine_in_Pyrimidine
Glutamate_Glutamine
Spermine_B-Alanine
phenylalanine_in_phenylalanine
Xanthine_Guanine
Aspartate_Aspartate-OUT
Hypoxanthine_Xanthine
CDP_Cytidine
IMP_XMP
F
MEBOCOST (G-S vs NC)
Prostaglandin E2
all-trans-Retinoic acid
Retinal
L-Methionine
L-Cysteine
Choline
L-Arginine
gamma-Aminobutyric acid
L-Glutamine
Geranylgeranyl-PP
myo-Inositol 1,4,5-trisphosphate
Heme
Farnesyl pyrophosphate
Adenosine triphosphate
D-Mannose
Dehydroascorbic acid
CAF-CAF
CAF-T
CAF-Tumor
Tumor-CAF
Tumor-T
Tumor-Tumor
T-CAF
T-T
T-Tumor
Cell-Cell Interaction Pair
log2FC

Figure S6. Supplemental Data to Figure 6 on Systematic Analysis of Cell-Cell Communication and Functional Alterations following the G-S therapy

(A) Heatmap depicting the differential cell-cell communication between the NC and G-S groups, in terms of interaction numbers and interaction strength. Red color indicates a stronger interaction number/strength in the G-S group.

(B, C) Differential cell-cell communication in specific signaling pathways between the NC and G-S groups: TGF-β signaling network (B) and TNF signaling network (C). In both networks, line thickness represents the strength of the cell-cell interactions observed in each group.

(D) KEGG pathway enrichment profiling of T cell transcriptomes, comparing the NC and G-S groups.

(E) KEGG pathway enrichment profiling of tumor cell transcriptomes, comparing the NC and G-S groups.

(F) Representative IHC images showing protein levels of HDAC3, FOSL1, JUNB, FASN, and PARP9 in the NC and G-S groups.

G-S, gemcitabine-SAHA.

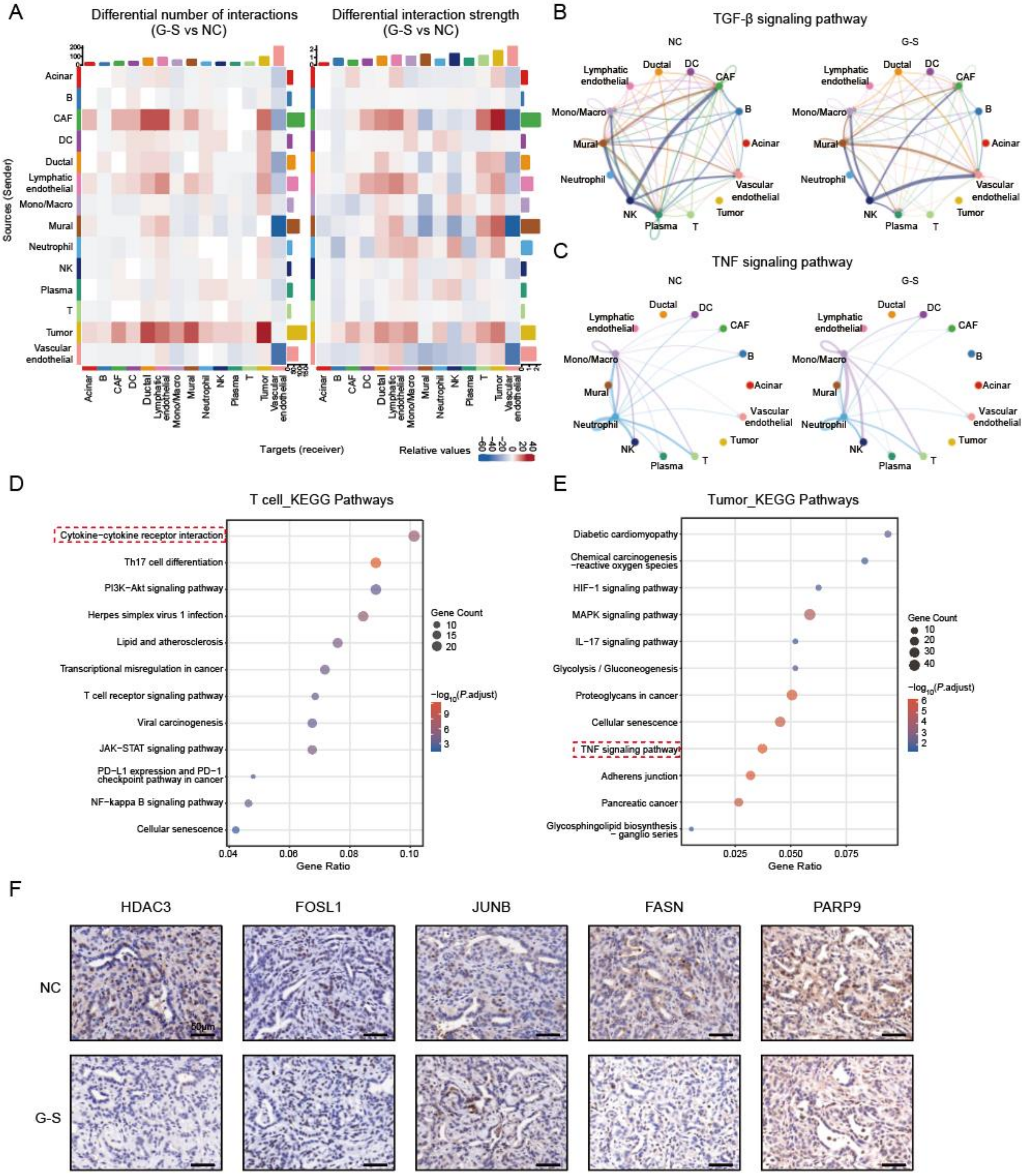
A
Differential number of interactions (G-S vs NC)
Differential interaction strength (G-S vs NC)
Sources (Sender)
Targets (receiver)
Relative values
Acinar
B
CAF
DC
Ductal
Lymphatic endothelial
Mono/Macro
Mural
Neutrophil
NK
Plasma
T
Tumor
Vascular endothelial
B
TGF-β signaling pathway
NC
G-S
C
TNF signaling pathway
NC
G-S
D
T cell_KEGG Pathways
Cytokine−cytokine receptor interaction
Th17 cell differentiation
PI3K−Akt signaling pathway
Herpes simplex virus 1 infection
Lipid and atherosclerosis
Transcriptional misregulation in cancer
T cell receptor signaling pathway
Viral carcinogenesis
JAK−STAT signaling pathway
PD−L1 expression and PD−1 checkpoint pathway in cancer
NF−kappa B signaling pathway
Cellular senescence
Gene Ratio
Gene Count
−log10(P.adjust)
E
Tumor_KEGG Pathways
Diabetic cardiomyopathy
Chemical carcinogenesis −reactive oxygen species
HIF−1 signaling pathway
MAPK signaling pathway
IL−17 signaling pathway
Glycolysis / Gluconeogenesis
Proteoglycans in cancer
Cellular senescence
TNF signaling pathway
Adherens junction
Pancreatic cancer
Glycosphingolipid biosynthesis − ganglio series
Gene Ratio
Gene Count
−log10(P.adjust)
F
HDAC3
FOSL1
JUNB
FASN
PARP9
NC
G-S
50μm

Figure S7. Supplemental Data to Figure 7 on Immune Cell Profiling in Humanized Mice and Clinical Survival Analysis

(A) Gating strategy for identifying human $CD45^{+}$ cells by flow cytometry in peripheral blood of humanized PDX mice.

(B) Gating strategy for analyzing tumor-infiltrating immune cells in tumor tissues from humanized PDX mice after treatment.

(C) Flow cytometry analysis of tumor-infiltrating human $CD8^{+}$ T cells and their PD-1 expression levels.

(D, E) Comparison of overall survival in “immune-hot” PDAC patients with high versus low expression of FASN (D) and PARP9 (E).

Data were represented as the mean ± SD. Statistical significance was determined by one-way ANOVA with Tukey’s post hoc tests (C) and by log-rank tests (D, E). **, p <0.01; ***, p <0.001; ****, p <0.0001; ns, not significant. GN, GEM-Nivolumab; GNS, gemcitabine-nivolumab-SAHA.

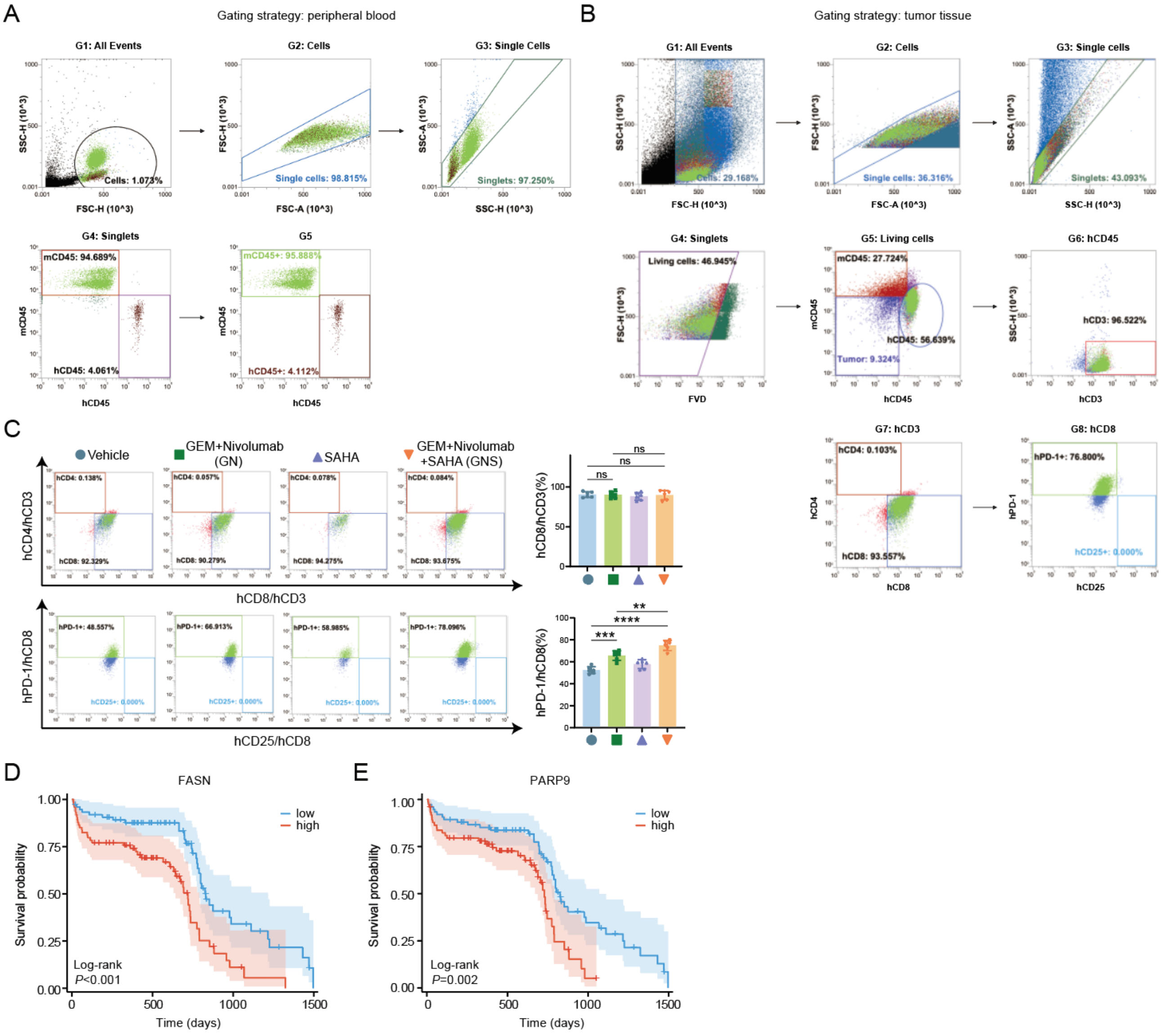
A
Gating strategy: peripheral blood
G1: All Events
Cells: 1.073%
G2: Cells
Single cells: 98.815%
G3: Single Cells
Singlets: 97.250%
G4: Singlets
mCD45: 94.689%
hCD45: 4.061%
G5
mCD45+: 95.888%
hCD45+: 4.112%
B
Gating strategy: tumor tissue
G1: All Events
Cells: 29.168%
G2: Cells
Single cells: 36.316%
G3: Single cells
Singlets: 43.093%
G4: Singlets
Living cells: 46.945%
G5: Living cells
mCD45: 27.724%
hCD45: 56.639%
Tumor: 9.324%
G6: hCD45
hCD3: 96.522%
G7: hCD3
hCD4: 0.103%
hCD8: 93.557%
G8: hCD8
hPD-1+: 76.800%
hCD25+: 0.000%
C
Vehicle
GEM+Nivolumab (GN)
SAHA
GEM+Nivolumab +SAHA (GNS)
hCD4/hCD3
hCD8/hCD3
hPD-1/hCD8
hCD25/hCD8
hCD8/hCD3(%)
hPD-1/hCD8(%)
D
FASN
E
PARP9
low
high
Survival probability
Time (days)
Log-rank P<0.001
Log-rank P=0.002

Table S1. Summary of cytokines.

Cytokine	Source	Identifier
TNF-α	MCE	Cat# HY-P7058
TNF-β	MCE	Cat# HY-P7304
IL-2Rα	MCE	Cat# HY-P7374
IL-18	MCE	Cat# HY-P70591
HGF	MCE	Cat# HY-P700604
FGF	MCE	Cat# HY-P70600A
IFN-γ	MCE	Cat# HY-P7025
CCL4	MCE	Cat# HY-P7257
CCL5	MCE	Cat# HY-P7282
IL-15	MCE	Cat# HY-P7371

Table S2. Summary of antibodies used in Western blot/CUT&tag.

Antibody	Source	Identifier
Anti-FOSL1	CST	Cat# D80B4
Anti-JUNB	Proteintech	Cat# 10486-1-AP
Anti-β-tubulin	Proteintech	Cat# 66240-1-Ig
Anti-GAPDH	Proteintech	Cat# 60004-1-Ig
Anti-FASN	Proteintech	Cat# 66591-1-Ig
Anti-PARP9	Proteintech	Cat# 17535-1-AP
Anti-Histone H3	CST	Cat# 4499
Anti-HDAC1	CST	Cat# 34589
Anti-HDAC2	CST	Cat# 57156
Anti-HDAC3	CST	Cat# 85057
Anti-Anti-Mouse Recombinant Secondary Antibody	Proteintech	Cat# RGAM001
Anti-Anti-Rabbit Recombinant Secondary Antibody	Proteintech	Cat# RGAR001
Anti-Anti-Rabbit IgG, Light Chain Specific	Proteintech	Cat# SA00001-7L

Table S3. Summary of antibodies used in Flow Cytometry.

Antibody	Source	Identifier
Zombie Aqua™ Fixable Viability Kit	BioLegend	Cat# 423102
Anti-mouse CD45	BioLegend	Cat# 103112
Anti-mouse CD3	BioLegend	Cat# 100222
Anti-mouse CD4	BioLegend	Cat# 100545
Anti-mouse CD8	BioLegend	Cat# 100731
Anti-mouse CD25	BD Pharmingen	Cat# 563061
Anti-mouse Ki-67	BioLegend	Cat# 652410
Anti-mouse IFN-γ	BioLegend	Cat# 505826
Anti-mouse Granzyme B	Thermo	Cat# 12-8898-82
Anti-mouse CD31	BioLegend	Cat# 102406
Anti-mouse Podoplanin	Thermo	Cat# 25-5381-82
Fixable Viability Dye	Thermo	Cat# 65-0866-14
Anti-human CD45	Thermo	Cat# 47-0459-42
Anti-mouse CD45	Thermo	Cat# 56-0451-82
Anti-human CD3	Thermo	Cat# 48-0037-42
Anti-human CD4	BioLegend	Cat# 317440
Anti-human CD8	BioLegend	Cat# 301031
Anti-human CD25	BioLegend	Cat# 356109
Anti-human CD279 (PD-1)	BioLegend	Cat# 379206

Table S4. Summary of antibodies used in IF/mIHC/IHC.

Antibody	Source	Identifier
Anti-HDAC3	CST	Cat# 3949
Anti-FOSL1	CST	Cat# 24814
Anti-JUNB	Proteintech	Cat# 10486-1-AP
Anti-CD8	Aifang	Cat# AFRM9067
Anti-Granzyme B	Aifang	Cat# AFRM0352
Anti-FASN	Proteintech	Cat# 66591-1-Ig
Anti-PARP9	Proteintech	Cat# 17535-1-AP
Anti-CK19	Aifang	Cat# AFRM0054
Anti-FAP	Huabio	Cat# ET1704-23
Anti-Collagen I	Aifang	Cat# AFRP0017
Anti-Fibronectin	Aifang	Cat# AFRM0355
Anti-α-SMA	Aifang	Cat# AFMM0002
Anti-FASN	Proteintech	Cat# 10624-2-AP
Anti-HDAC3	Proteintech	Cat# 81211-1-RR
Anti-FOSL1	CST	Cat# 28801
